# Erasable FAP Targeted Spray‐On Probe for Fluorescence‐Guided Surgery

**DOI:** 10.1002/advs.202523986

**Published:** 2026-03-24

**Authors:** Zachary Rabinowitz, Riley J. Deutsch‐Williams, Ralph Weissleder

**Affiliations:** ^1^ Center for Systems Biology Massachusetts General Hospital Boston Massachusetts USA; ^2^ Department of Systems Biology Harvard Medical School Boston Massachusetts USA

**Keywords:** cancer, FAP, fibroblast, fluorescence, rhodamines, surgery

## Abstract

Fluorescence‐guided surgery is an emerging clinical field that aims to improve cancer detection in real‐time, enabling spot‐on therapeutic decisions. The use of fluorescent affinity probes has primarily involved systemically injected probes and, more recently, “spray‐on probes”. A current challenge has been to develop “turn‐off” probes that can be inactivated by a UV light pulse and reduce background noise during iterative decision‐making. Here, we report on the first development of a FAP‐targeted erasable spray‐on probe (ESOP). The probe consists of three components: (i) a highly bright, yellow light‐emitting rhodamine dye, (ii) a photo‐responsive linear triazene linker, and (iii) a FAP affinity ligand. We demonstrate that this probe could enable highly sensitive tumor‐specific margin visualization within minutes after a spray‐on application. Moreover, we show that the fluorescent signals could be fully erased with a 2‐min UV‐light pulse in tissue, permitting iterative staining applications with consistently high tumor‐to‐background ratios (TBRs). ESOP targeted against cancer or host tissue is feasible and is likely to yield transformative impacts on next‐generation cancer surgeries, ultimately improving patient care.

## Graphical abstract

We present the first erasable spray‐on probe for fluorescence‐guided surgery. Following topical delivery, FAP‐FLASH550 targets fibroblast activation protein–expressing stromal cells, delineating tumor margins with high tumor‐to‐background ratios. A 365 nm UV light pulse rapidly erases fluorescent signals through triazene photodegradation and subsequent rhodamine spirocyclization, enabling iterative stain–erase–restain cycles that preserve high tumor contrast across consecutive resections.

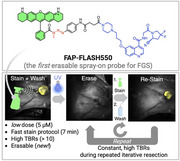

## 1 Introduction

The field of fluorescence‐guided surgery (FGS) has experienced tremendous advancements over the last two decades, slated to become an indispensable tool in future surgeries. Fluorescence imaging utilizes a combination of fluorescent imaging probes and specialized detection systems to visualize cancers, their margins, and adjacent vital structures with greater accuracy. The use of indocyanine green (ICG) dates back to the 1960s, while fluorescent molecular imaging probes have been developed over the last 25 years [1, 2, 3, 4]. Despite these advancements, significant challenges remain in FGS as evidenced by recent clinical trials [5]: even with molecular agents, the false negative and false positive rate remains high, largely due to low tumor‐to‐background ratios (TBRs), complex administration requirements, and the not insignificant side effect rate due to systemic administration. There is thus a need for “on demand” probes that achieve a much higher TBR, resulting in more accurate margin detection. We have recently developed “spray‐on probes” (SOP) that can significantly enhance fluorescence‐guided surgery, detect smaller tumors (higher TBRs), enable new functionalities, improve imaging accuracy and speed, and reduce the dose [6, 7]. While highly useful, these probes were “always‐on” and required extensive washing steps to remove non‐specific signals from surrounding tissues for repeated SOP applications to the surgical area, reducing TBRs over multiple applications. We thus reasoned that probes that could be turned “off” on demand would be an ideal solution.

Here we describe the synthesis of an erasable FAP‐targeted fluorescent imaging probe, referred to as FAP‐FLASH550, whose fluorescence at 570 nm (λ_ex_, 550 nm) can be erased by a quick UV‐light pulse. The design of the probe is based on our previous development of “FLASH‐off” rhodamines [8], which feature a photo‐immolating linear triazene‐rhodamine dye conjugate. After photolysis, the triazene is degraded, releasing nitrogen gas and the methyl xanthamide, undergoes irreversible spirocyclization, rendering the dye in a closed, non‐fluorescent form. We show that after exposure to a pulse of UV‐light, the probes’ fluorescence emission at 570 nm decreases ∼2000‐fold in under 10 s in aqueous buffer. Furthermore, we show that the probe exhibits high stability in aqueous buffer and is turned “off” solely by irradiation with UV‐light. We then applied the probe in live mammary tumor‐bearing mice to assess its performance for FGS. We demonstrated that the probe could provide sensitive tumor‐specific detection using a 7‐min staining protocol and maintain consistent, superb TBRs (>10) across iterative applications in an intraoperative setting.

## 2 Results and Discussion

### 2.1 Design and Synthesis

Our goal was to develop an “erasable” SOP (ESOP) to improve sensitive detection of tumor margins during FGS, compared to “always‐on” SOPs. Previously, we described “always‐on” SOPs targeting FAP [6, 7] for topical application to augment tumor margin detection during oncological surgery. However, we observed that FGS with these “always‐on” SOPs could be further improved if one could reduce the background arising from normal tissue during repeat cycles of washing and re‐staining (Figure S1A). We hypothesized that an “erasable” FAP‐targeted compound could be developed, enabling background reduction during iterative staining (Figure S1B). Because intraoperative imaging is a continuous process in a changing landscape till the entirety of a tumor is removed, residual stains from prior administrations can lead to a high background. Our approach with a 2‐min UV pulse solves this problem.

**FIGURE 1:**
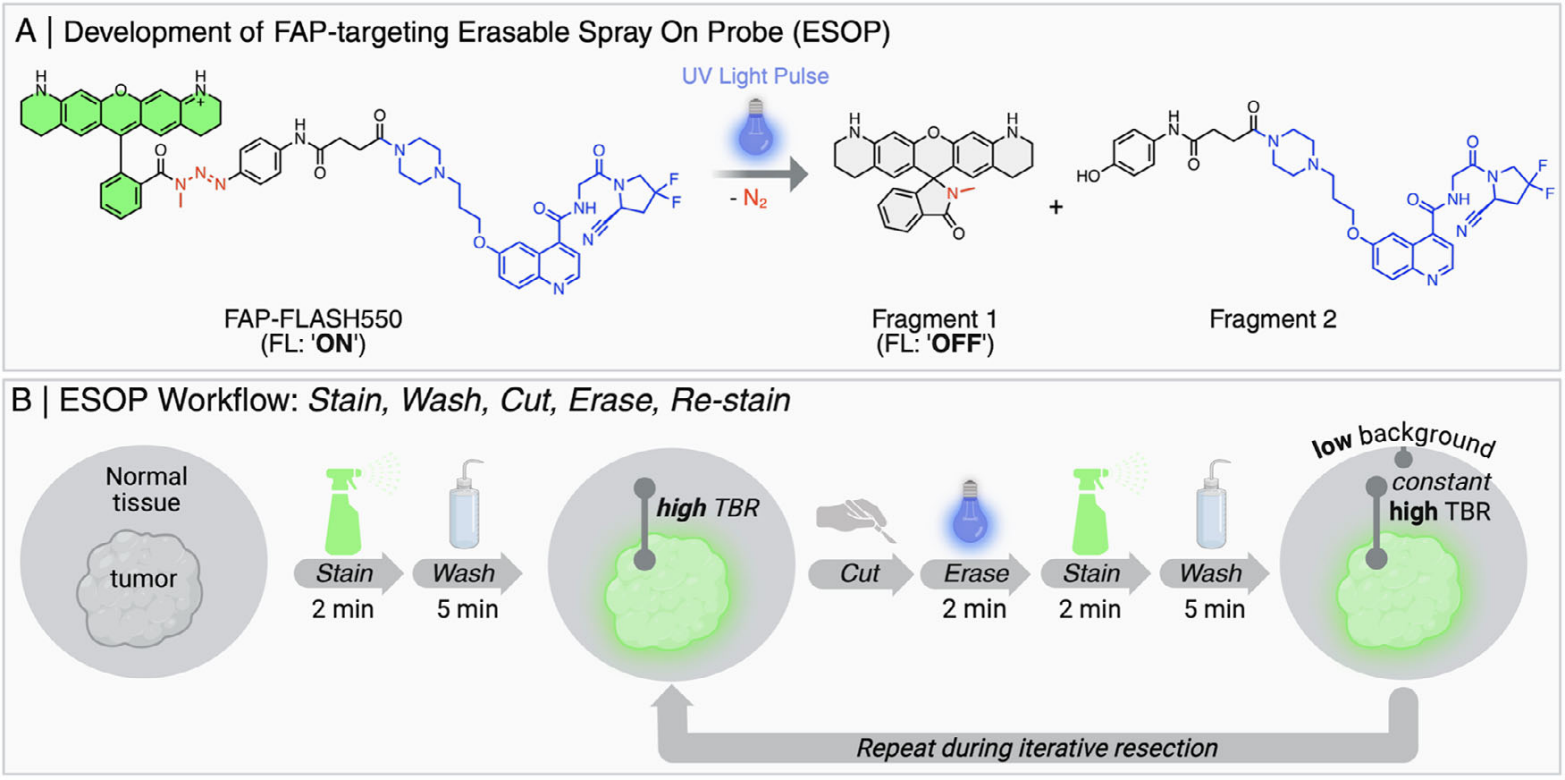
Mechanism and application of FAP‐FLASH550. A) Structure of FAP‐FLASH550 and its reaction with a UV light pulse. In the presence of a UV light pulse, the triazene moiety decomposes, releasing nitrogen gas, and generating two fragments: the closed, non‐fluorescent methyl xanthamide (fragment 1) and the FAP binding moiety‐based fragment (fragment 2). B) After a short topical application procedure (2 min stain after a “spray” of probe solution, followed by a 5 min aqueous wash), ESOPs could stain tumor margins with highly sensitive tumor‐to‐background ratios (TBRs). Then, guided by specific fluorescent signals, tumors could be resected, followed by erasure of any remaining fluorescent signals via a 2‐min UV pulse. Then, the operating field could be re‐stained to re‐inspect tumor margins with high sensitivity. This workflow could be repeated during iterative resections, as needed.

The new ESOP molecule (FAP‐FLASH550) consists of three components: (i) a very bright rhodamine dye [9], (ii) a photo‐cleavable triazene [8], and (iii) a high‐affinity small‐molecule FAP inhibitor (UAMC1110) (Figure 1A). To erase fluorescent signals arising from the probe, a UV light pulse is applied, resulting in the photolytic cleavage of the triazene moiety and the generation of nitrogen gas. Consequently, two fragments are produced: the closed, non‐fluorescent methyl xanthamide (fragment 1) and FAP‐binding moiety‐based fragment (fragment 2). The fluorescent signals arising from the rhodamine dye are terminated due to irreversible, intramolecular spirocyclization of the methyl xanthamide. During FGS, we envisioned that FAP‐FLASH550 could be applied on‐demand as a spray to suspected tumor‐bearing tissue to provide sensitive tumor visualization (high TBR) via targeting FAP‐expressing cancer‐associated fibroblasts (CAFs) within the tumor stroma (Figure 1B). Then, guided by specific fluorescent signals, the tumor could be resected, followed by an erasure step to remove any remaining fluorescent signals via a 2‐min UV pulse, and then re‐stain to re‐inspect tumor margins with high sensitivity. This workflow could be repeated during iterative resections, as needed.

**FIGURE 2:**
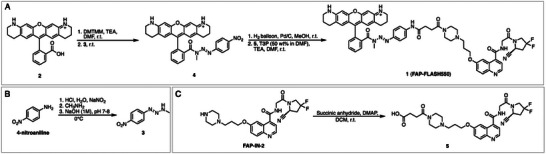
Synthetic route to FAP‐FLASH550. A) Two‐step synthetic route to obtain compound 1 (FAP‐FLASH550) starting from rhodamine compound 2. B) Synthesis of the triazene linker 3 from 4‐nitroaniline. C) Synthesis of compound 5.

The synthesis of FAP‐FLASH550 is presented in Figure 2. We commenced the synthesis via conjugation of previously synthesized compounds, rhodamine 2 [9] with triazene 3 [8, 10], using the coupling reagent DMTMM in the presence of TEA in DMF to yield compound 4. Then, compound 4 was subjected to Pd/C‐catalytic hydrogenation with H_2_ gas to afford the aniline intermediate, which was used immediately without further purification for the next step. In parallel, to conjugate FAP‐IN‐2 to the aniline intermediate, we installed a carboxylic acid handle on the piperazine moiety using succinic anhydride in the presence of DMAP in DCM to yield compound 5. Lastly, the aniline intermediate was conjugated to compound 5 using T3P‐mediated amidation in the presence of TEA in DMF to successfully generate the desired probe, FAP‐FLASH550 (compound 1) in 16% yield and in excellent purity (>95% pure). The successful generation of each compound was verified using high‐performance liquid chromatography‐mass spectrometry (HPLC‐MS). HPLC traces and mass spectra can be found in Figure S2. All observed masses were consistent with calculated values. Furthermore, ^1^H nuclear magnetic resonance (^1^H NMR) spectroscopy was used to confirm the structures (Figures S3–S7).

### 2.2 Evaluation of Fluorescence and Photochemical Properties of ESOP

**FIGURE 3:**
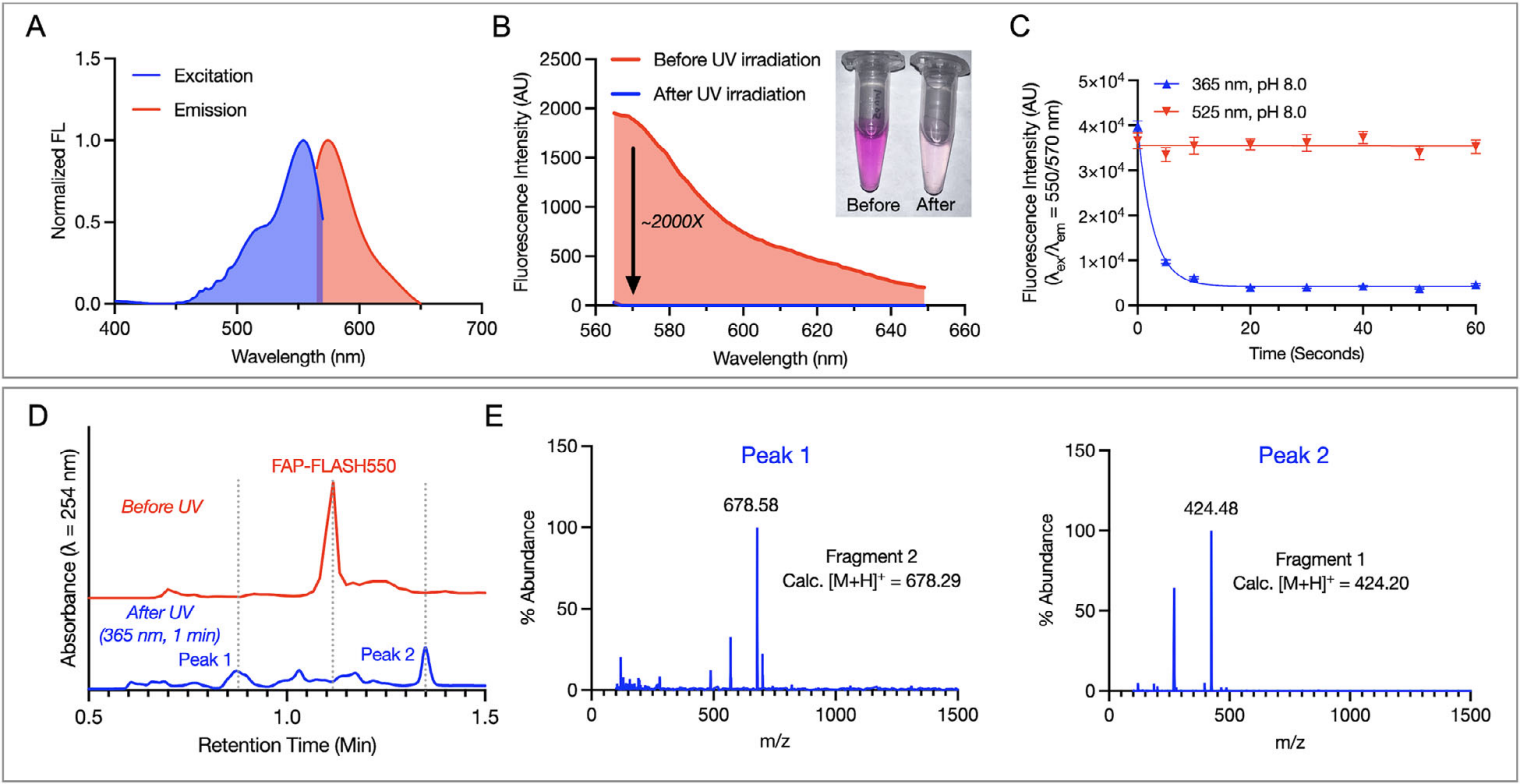
Evaluation of photolysis of FAP‐FLASH550. A) Fluorescence excitation and emission spectra for FAP‐FLASH550 (1 µm). B) Fluorescence spectra of FAP‐FLASH550 (50 µm) before and after exposure to a UV light pulse (365 nm, 1 min) in phosphate‐citrate buffer (pH, 8). Inset: Pictures of eppendorf tubes containing solutions of FAP‐FLASH550 before and after UV irradiation. C) Fluorescence quenching of FAP‐FLASH550 (5 µm) using UV light (365 nm) or green light (525 ± 36 nm) in phosphate‐citrate buffer (pH, 8) over 1 min. Exposure to green light yielded no observable fluorescence quenching, whereas irradiation with UV light led to fast fluorescence quenching. D) HPLC traces of FAP‐FLASH550 (50 µm) before and after UV irradiation (365 nm, 1 min) in phosphate‐citrate buffer (pH, 8). E) Mass spectra (ESI) of peak 1 and peak 2 present in (D).

With the probe in‐hand, we first examined the fluorescence excitation and emission spectrum of FAP‐FLASH550. The probe exhibited a fluorescence excitation and emission maxima at 550 and 570 nm, respectively (Figure 3A). Then, we assessed the fluorescence stability of FAP‐FLASH550 in aqueous buffer at relevant pH values to physiological conditions and staining protocols (pH, 6.8, 7.4, and 8) over a long‐incubation period. As shown in Figure S9A, the fluorescent signals were stable, confirming the absence of non‐specific hydrolysis of the probe, which would result in potential fluorescence quenching (Figure S9B). After confirming the probe's robust stability in mildly basic, aqueous buffer, we sought to assess the fluorescence quenching of FAP‐FLASH550 in response to brief pulses of UV light (365 nm, 2.05 mW/cm^2^) in phosphate‐citrate buffer (pH 8). After exposing a solution of FAP‐FLASH550 (50 µm) with UV light for 1 min, we observed a significant decrease (∼2000‐fold) in fluorescence emission at 570 nm, which correlated with a noticeable loss of dark pink color of the solution (Figure 3B).

Next, we determined the kinetics of UV light‐mediated fluorescence quenching of FAP‐FLASH550. For the erase step in intraoperative applications, it is essential that fluorescence quenching occurs fast, so as to not significantly prolong the application procedure during tumor surgery. Thus, we irradiated solutions of probe (5 µm) in phosphate‐citrate buffer (pH, 8) with pulses of UV light (365 nm, 2.05 mW/cm^2^) for various durations (0–60 s) and then, measured the fluorescence intensities at 570 nm (λ_ex_, 550 nm) (Figure 3C). We experimentally observed that the reaction proceeded extremely fast with an average half‐life of 1.9 s. In parallel, we irradiated probe (5 µm) solutions to green light using the lamp of our home‐built FGS imaging system in combination with a TRITC filter (λ_ex_, 525 ± 36 nm) to assess the fluorescence stability under the fluorophores’ excitation source. After exposure to green light (λ_ex_, 525 ± 36 nm), the fluorescence signals were stable (Figure 3C). This confirms two important considerations for ESOPs: (I) UV light is solely responsible for fluorescence quenching and (II) the probes’ fluorescent signals are stable in the presence of the FGS imaging systems’ excitation source. Additionally, we repeated this experiment in phosphate‐buffered saline (pH, 7.4) and the results were similar, confirming that the fast fluorescence quenching kinetics can also occur at physiological pH (Figure S10).

To better understand the photolysis reaction mechanism, the reaction solution of FAP‐FLASH550 (50 µm) was exposed to a 1‐min UV light pulse (365 nm, 2.05 mW/cm^2^) in phosphate‐citrate buffer (pH, 8) and then, subjected to HPLC‐MS analysis. As shown in Figure 3D, after exposure of FAP‐FLASH550 with UV light, complete conversion of the probe occurred within 1 min, generating two new peaks: peak 1 and peak 2. We obtained the mass spectrums at the relevant retention times to tentatively assign the structures of these two new peaks. As seen in Figure 3E, the observed mass‐to‐charge ratio values (m/z, electrospray ESI) of peak 1 (retention time, 0.87 min) and peak 2 (retention time,1.35 min) were consistent with the calculated m/z values for fragment 2 and fragment 1, respectively. This is consistent with the proposed photolytic reaction mechanism [8], where UV light catalyzes the cleavage of the triazene moiety, resulting in the release of nitrogen gas, followed by spirocyclization of the methyl xanthamide fragment 1, terminating fluorescent signals. To investigate the pH‐dependent fluorescence emission of fragment 1, we first synthesized the rhodamine‐based compound via a two‐step, one‐pot reaction (Figure S11A). Successful formation of fragment 1 was confirmed by HPLC‐MS (Figure S2E,J) and ^1^H NMR spectroscopy (Figure S8). Solutions of fragment 1 (5 µm) were prepared in buffers ranging from pH 3 to 9, and fluorescence was measured at 570 nm (λ_ex_, 550) (Figure S11B). Fragment 1 was non‐fluorescent at pH >7, with fluorescence increasing under acidic conditions, consistent with pH‐dependent spirocyclization: the open, fluorescent form predominates under acidic conditions, while the closed, colorless spirolactam form is favored under basic conditions (Figure S11C). Because the surgical field is typically pH ∼7.4–8.0, Fragment 1 is expected to remain non‐fluorescent under these conditions, enabling fluorescence erasure after UV irradiation.

In summary, FAP‐FLASH550 fluoresces in the yellow region of the visible spectrum (λ_ex/_λ_em,_ 550/570 nm) and displays robust stability in aqueous buffer. Importantly, we demonstrated that the probe exhibits extremely fast fluorescence quenching kinetics at both physiological and mildly basic pH solely by UV light irradiation, making it highly suitable as an ESOP. Therefore, we next investigated the performance of FAP‐FLASH550 as an ESOP for highly sensitive tumor‐specific margin detection.

### 2.3 Testing of FAP‐Targeting ESOP in Tissue

Before exploring the performance of FAP‐FLASH550 in tumor tissue, we initially assessed its binding affinity to murine FAP (mFAP) using the fluorogenic Z‐Gly‐Pro‐AMC assay. As shown in Figure S12, FAP‐FLASH550 showed remarkable binding affinity to mFAP in the low nanomolar range (IC_50_, 3 nm). Furthermore, this remarkable FAP‐binding affinity is similar to other FAP imaging probes [7, 11, 12]. Also, we assessed the cytotoxicity of FAP‐FLASH550 using NIH/3T3 fibroblast cells. Briefly, cells were incubated with varying concentrations of FAP‐FLASH550, after which the probe‐contained media was replaced with fresh media and cells were cultured for an additional 3 days. Cell viability was then evaluated using PrestoBlue, with fluorescence intensity measured at 590 nm (λ_ex_ = 560 nm) following a 60 min incubation. As shown in Figure S13, cell viability remained above 90% across all tested probe concentrations, indicating that FAP‐FLASH550 exhibits minimal cytotoxicity under these conditions. We next optimized the tissue staining protocol to achieve an optimal TBR (TBR > 5) in a timeframe conducive to surgery by optimizing stain time and probe concentration, while keeping a standard 5‐min PBS wash. Briefly, ex vivo 4T1 tumor tissue sections or gonadal fat pad sections (300 µm) were stained for variable amounts of time (2, 5, or 10 min) with various probe concentrations (0.5, 1, 5, and 10 µm), followed by imaging with a widefield fluorescence imaging system. Mean intensity values for tumor and fat tissues were measured using ImageJ and used to calculate TBRs. As shown in Figure S14, we experimentally determined that with a 2‐min stain time at a concentration of 5 µm, followed by a 5‐min PBS wash, tumor contrast was highest. This 7‐min topical staining protocol for tumor‐margin detection is consistent with our previous FAP‐targeting SOPs [6, 7]. We hypothesized that ESOPs are superior to “always‐on” SOPs for intraoperative tumor imaging. ESOPs enable the erasure of fluorescent signals after tumor resection, allowing residual stains to be removed prior to repeated administrations to ensure consistently high TBRs across multiple applications. Moreover, compared to systemically administered FGS agents, SOPs allow much higher TBRs and have been shown to detect even microscopic cancer deposits.

**FIGURE 4:**
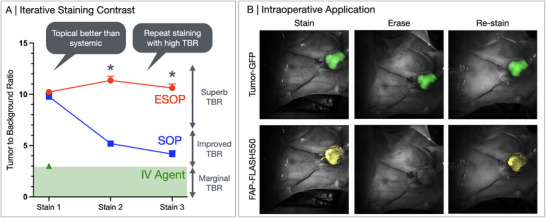
ESOPs enable higher TBRs than SOPs and IV Agents. A) After iterative staining (*n* = 3), ESOP yielded consistently higher TBRs than SOP. Additionally, ESOP produced superior tumor contrast than systemically administered IV agent (* = *p* < 0.05) (repeated measures ANOVA with Tukey's Posthoc Test). B) Intraoperative application of FAP‐FLASH550 (5 µm) was performed on an orthotopic 4T1‐Venus GFP mammary tumor in a murine model. After spray‐on application of the ESOP, white light (gray), tumor‐GFP (green) and FAP‐FLASH550 (yellow) fluorescence images were acquired (stain). The operative field was then exposed to a 2‐min pulse of UV light (365 nm) and then, re‐imaged (erase). The FAP‐FLASH550 channel showed complete erasure of fluorescent signals within 2 min. Subsequently, the operative field was re‐stained with FAP‐FLASH550 to show iterative staining could be achieved (re‐stain).

To confirm this observation, we performed a proof‐of‐concept experiment in ex vivo tissue to validate that an “erase and re‐stain” protocol yields consistent high TBRs, compared to a “re‐stain only” procedure – a limitation of “always‐on” SOPs (Figure 4A). Briefly, ex vivo 4T1 tumor tissue sections or gonadal fat pad sections (300 µm) were exposed to a 2‐min stain with FAP‐FLASH550 (5 µm), followed by a 5‐min PBS wash. After imaging, tissue sections were either exposed to a 2‐min UV light pulse (365 nm, 2.05 mW/cm^2^) or kept away from UV light. Then, tissue sections were re‐stained following the same 7‐min procedure and re‐imaged (Figure S15). This process was repeated thrice and mean intensity values were measured and TBRs were calculated. Remarkably, with an “erase and re‐stain” protocol after each stain, we observed superb, consistently high TBRs across iterative topical applications (TBR ≈ 10) (*n* = 3) (Figure 4A). However, with the “re‐stain only” protocol, there was a ∼50% decrease in TBR after re‐staining. This suggests that after iterative applications of an “always‐on” SOP, the TBR could decrease significantly due to accumulation of non‐specific signals in surrounding non‐tumor tissue (e.g., fat) after each repeated application. Moreover, to compare the TBR of SOPs with systemically administered FGS agents, we administered FTF‐Cy5 [7] intravenously (200 µg) via tail vein in 4T1 Venus‐GFP tumor‐bearing BALB/C mice. After 1 h, tumors and adjacent mammary tissue were resected and imaged using an epifluorescence microscope (Figure S16). The mean intensity values for tumor and nearby mammary fat pad were measured and used to calculate TBRs (Figure 4A). We observed a marginal TBR of ∼3, which is consistent with other systemically administered FAP‐targeting probes [13, 14, 15] and other clinically approved FGS agents [16, 17, 18]. This suggests that ESOPs could provide significantly higher TBRs and thus, higher tumor conspicuity than both “always‐on” SOPs and systemically administered FGS agents.

### 2.4 Performance of FAP‐Targeting ESOP in an Intraoperative Setting

As we confirmed that the ESOP can provide high TBRs in tumor sections ex vivo, we next sought to demonstrate the “stain‐erase‐restain” protocol using FAP‐FLASH550 in the more complex intraoperative setting in live mammary tumor‐bearing mice. On the day of surgery, the abdominal skin above the right mammary tumor was removed to expose the tumor‐bearing gland of a 4T1 Venus‐GFP tumor. Following our previous “spray‐on” procedure in intraoperative settings [6, 7], a calcium chloride solution (3% w/v) was first sprayed and then, a solution of FAP‐FLASH550 (5 µm) in alginate (0.5% w/v in ddH_2_O) was sprayed onto the operative field, which resulted in a probe‐consisting gel that was uniformly formed across the tissue surface. After a 2‐min stain, the gel was washed off using a 1‐min wash with EDTA solution (3% w/v) to depolymerize the cross‐linked alginate gel, followed by a 4‐min wash with PBS to remove non‐specific staining. Using a home‐built FGS imaging system, white light reflectance (gray) and fluorescence images in both the tumor‐GFP (green) and FAP‐FLASH550 (yellow) channels were acquired. As shown in Figure 4B, in the first stain, the location of the tumor marked by tumor‐GFP signal is concordant with FAP‐FLASH550 signal, suggesting successful achievement of tumor‐specific margin detection via targeting FAP‐expressing CAFs in the tumor stroma. Then, the surgical site was exposed to a 2‐min pulse of UV light (365 nm, 2.05 mW/cm^2^) and then re‐imaged. We observed complete erasure of fluorescent signals arising from FAP‐FLASH550, as reflected by a decrease in the TBR from ∼10 to 1 following brief UV irradiation (Figure S17). Importantly, the use of UV‐A light for intraoperative applications may be safe, as the power density employed in this study (2.05 mW/cm^2^) is negligible relative to the irradiance produced by standard operating room lighting, which typically remains on for extended periods during surgical procedures [19]. Moreover, this level of UV‐A exposure is comparable to that of a Wood's lamp, a device widely regarded as safe for human use and routinely used in clinical settings for diagnostic purposes [20, 21]. After erasure, we then performed a re‐stain of the surgical site with FAP‐FLASH550 and again, observed sensitive tumor‐specific margin detection. We next optimized the kinetics of in vivo fluorescence erasure of FAP‐FLASH550 signals from tumor using UV light. Following spray‐on application of FAP‐FLASH550 to the surgical site, staining was visualized by fluorescence imaging (Figure S18A) and tumor‐specific labeling was confirmed by histological analysis (Figure S19). The tissue was exposed to UV light pulse (365 nm, 2.05 mW/cm^2^) in 10‐s intervals between image capture. Time‐lapse imaging after initial tumor staining showed complete fluorescence erasure within 50 s, demonstrating the rapid kinetics of fluorescence erasure via the triazene‐based mechanism (Figure S18B). Additionally, we found that the erased probes were non‐fluorescent at biologically relevant pH values. Tumors stained with FAP‐FLASH550 and exposed to a UV light pulse maintained consistently low fluorescence intensity when sequentially incubated in solutions at pH 8, 6.5, and 4.5 after exposure UV (Figure S20). These results suggest that a FAP‐targeting ESOP can provide high tumor‐margin conspicuity after a short staining protocol (7 min) and be erased on tissue with a short UV light pulse to provide consistently superb TBRs across multiple topical applications. Overall, the staining protocol plus the erasure step is still under 10 min, allowing on‐demand “spray‐on” application of ESOPs during tumor surgery.

## 3 Conclusion

In this study, we have developed FAP‐FLASH550, the first FAP‐targeting ESOP for fluorescence‐guided cancer surgery. We show that this probe is stable in aqueous buffer and fluorescent signals could be quenched (∼2000‐fold) within seconds using a pulse of UV light. Moreover, this probe could be applied within minutes onto the operating field, providing highly sensitive tumor‐specific margin detection in live tumor‐bearing mice. Additionally, we demonstrate that fluorescent signals could be erased rapidly to enable highly reliable repeated tumor‐specific staining of the surgical field after tumor resection. Thus, the development of UV‐light erasable spray‐on probes based on a photo‐reactive triazene‐rhodamine dye conjugate is technically feasible and straightforward. Additional steps are required for future clinical translation under a physician‐sponsored investigational new drug (IND) application. This includes comprehensive preclinical toxicity studies and optimization of dosing and application protocols for human use. We believe that ESOPs will transform next‐generation fluorescence‐guided cancer surgeries and ultimately improve the rate of negative margins and patient care.

## 4 Materials and Methods

### 4.1 Materials

All reagents were purchased from commercial sources (Sigma‐Aldrich, AmBeed) and used without further purification. Solvents were obtained from Sigma–Aldrich and deuterated solvents were purchased from Cambridge Isotope Laboratories.

### 4.2 Purification

Compounds were purified using a Biotage Sfär C18 D Duo 100 Å 30 µm 6 g column (Biotage, Cat. FSUD‐0401‐0006) on a Buchi Pure C‐850 FlashPrep with a linear binary gradient solvent system. Two different solvent systems were used for purification, depending on the molecule. Solvent system 1: water with 0.1% formic acid (solvent A) and acetonitrile with 0.1% formic acid (solvent B). Solvent system 2: ammonium formate (2.5 mm, pH 8) (solvent A) and acetonitrile (solvent B). Reverse‐phase high‐pressure liquid chromatography (RP‐HPLC) was performed using the following linear gradient: 2% solvent B (0–5 min), 2%–80% solvent B (5–20 min), 80% solvent B (20–25 min) at flow rate of 20 mL per min.

### 4.3 Characterization

High‐performance liquid chromatography‐mass spectrometry (HPLC‐MS or LCMS) analysis was performed on a Waters instrument equipped with a Waters 2424 ELS detector, Waters 2998 UV‐VIS diode array detector, and Waters 3100 ESI‐MS module (LRMS) with an Xterra MS C18 column (125 Å, 4.6 × 50 mm column). Separations employed a linear binary gradient solvent system: water with 0.1% formic acid (solvent A) and acetonitrile with 0.1% formic acid (solvent B) at flow rate of 5 mL per min. The method was as follows: 5%–100% solvent B (0–1.5 min), 100% solvent B (1.5–2 min).

NMR spectra were recorded on a Bruker Avance Ultrashield 400 MHz spectrometer. ^1^H NMR chemical shifts are reported in ppm and referenced internally with respect to residual protons (𝛿, 2.50 for DMSO‐d6; 4.87 for CD_3_OD). Coupling constants are reported in Hz.

### 4.4 Synthetic Procedures

#### 4.4.1 Compound 2

Compound 2 was synthesized following a previously reported protocol [9]. In brief, a mixture of 7‐hydroxy‐1,2,3,4‐tetrahydroquinone (2.0 g, 13 mmol, 1 eq.) and phthalic anhydride (3.0 g, 20 mmol, 1.5 eq.) was heated at 170°C for 3 h using an oil bath. After 3 h, the reaction was allowed to cool to room temperature. Then, an additional portion of 7‐hydroxy‐1,2,3,4‐tetrahydroquinone (2.0 g, 13 mmol, 1 eq.) and H_3_PO_4_ (85 wt.% in H_2_O) (6.5 mL) were added, and the reaction was placed at 170°C for another 3 h. Subsequently, the reaction was cooled and methanol (40 mL) was added. The reaction was refluxed for several minutes, cooled, and diluted with dichloromethane (80 mL). The precipitate was filtered off and dried in vacuo to afford a dark purple solid (Yield: 3.6 g, 44%). ^1^H NMR (400 MHz, [D_6_]DMSO, ppm): δ = 8.17 (d, J = 7.7 Hz, 1H), 7.78 (dtd, J = 23.4, 7.5, 1.4 Hz, 2H), 7.35 (d, J = 7.5 Hz, 1H, H‐8), 6.64 (s, 2H, H‐1), 6.57 (s, 2H, H‐5), 3.35 (t, J = 5.6 Hz, 4H, H‐4), 2.60 (dd, J = 8.0, 5.0 Hz, 4H, H‐2), 1.82 – 1.68 (m, 4H, H‐3).

#### 4.4.2 Compound 3

Compound 3 was synthesized following a previously reported protocol [8]. In brief, 4‐nitroaniline (300 mg, 2.17 mmol, 1 eq.) was dissolved in 1 M HCl (15 mL). The brown‐yellow solution was cooled to 0°C using an ice‐bath. Then, NaNO_2_ (330 mg, 4.78 mmol, 2.2 eq.) in H_2_O (5 mL) was added drop‐wise to the reaction solution. The reaction was allowed to stir for 1 h at 0°C and then, methylamine (2 M in THF) (2.17 mL, 4.734 mmol, 2 eq.) was added drop‐wise. After 5 min, 1 M NaOH (5 mL) was added until the pH of the solution reached ∼7–8 to yield a brownish‐yellow precipitate, which was collected by filtration and dried under vacuum at room temperature overnight (Yield: 306 mg, 78%). The product was used without further purification. The spectral characteristics were analyzed and determined to be consistent with literature values [8]. ^1^H NMR (400 MHz, [D_6_]DMSO, ppm): δ 8.40 (d, J = 8.7 Hz, 1H), 8.31 (d, J = 8.7 Hz, 1H), 7.92 (d, J = 8.6 Hz, 1H), 7.71 (m, 1H), 3.33 (s, overlapping signals, 3H).

*Note*: The authors suggest avoiding further purification of the crude material or recrystallization in ethanol or benzene. The crude was found to be relatively stable when stored dry, under argon at −20°C for no longer than 2 weeks.

*Caution*: Monomethyl triazenes can be potential carcinogens and careful handling in a fume hood is highly advised.

#### 4.4.3 Compound 4

To a solution of compound 2 (313 mg, 0.760 mmol, 1 eq.) dissolved in anhydrous dimethylformamide (DMF) (3 mL), 4‐(4,6‐Dimethoxy‐1,3,5‐triazin‐2‐yl)‐4‐methylmorpholinium chloride (DMTMM) (421 mg, 1.52 mmol, 2 eq.) and triethylamine (TEA) (212 µL, 1.52 mmol, 2 eq.) were added. The reaction was allowed to stir for 1 h. Then, compound 3 (274 mg, 1.52 mmol, 2 eq.) in DMF (1 mL) was added drop‐wise and the reaction was allowed to stir overnight. The reaction was immediately purified by RP‐HPLC (solvent system 2 was used, see Purification section) and lyophilized to yield the product as a pink powder (Yield: 23 mg, 5%). ^1^H NMR (400 MHz, [D_6_]DMSO, ppm): δ = 8.34 (d, J = 8.6 Hz, 2H), 8.00 – 7.72 (m, 4H), 7.53 (d, J = 8.5 Hz, 3H), 6.67 (s, 4H), 3.19 (s, 3H), 2.46 – 2.34 (m, 5H), 2.17 (d, J = 16.4 Hz, 2H), 1.71 – 1.55 (m, 6H). MS (ESI): m/z calcd for C_33_H_29_N_6_O_4_^+^: 573.22 [M]^+^, found: 573.58.

#### 4.4.4 Compound 5

To a solution of FAP‐IN‐2 (20 mg, 0.041 mmol, 1 eq.) in anhydrous DCM (1 mL), DMAP (5 mg, 0.041 mmol, 1 eq.) and Succinic anhydride (6.2 mg, 0.062 mmol) were added. The reaction was allowed to stir for 30 min. The reaction was then evaporated in‐vacuo and then, redissolved in dimethylsulfoxide (DMSO) (500 µL) prior to purification. The reaction was purified by RP‐HPLC (solvent system 1 was used, see Purification section) and lyophilized to yield the product as a white powder (Yield: 20 mg, 83%). ^1^H NMR (400 MHz, CD_3_OD, ppm): δ = 8.72 (d, J = 4.5 Hz, 1H), 7.98–7.88 (m, 2H), 7.53 (d, J = 4.4 Hz, 1H), 7.43 (dd, J = 9.2, 2.7 Hz, 1H), 5.09 (dd, J = 9.3, 3.2 Hz, 1H), 4.28 (q, J = 4.6 Hz, 3H), 3.65 (d, J = 5.9 Hz, 4H), 3.28 (p, J = 1.6 Hz, 2H), 2.82 (ddd, J = 33.1, 19.1, 6.2 Hz, 7H), 2.62 (s, 5H), 2.16 (d, J = 7.8 Hz, 2H). MS (ESI): m/z calcd for C_28_H_32_F_2_N_6_O_6_: 587.24 [M+H]^+^, found: 587.86.

#### 4.4.5 Compound 1 (FAP‐FLASH550)

In a 25 mL round‐bottom flask, compound 4 (19 mg, 0.033 mmol, 1 eq.) was dissolved in anhydrous methanol (2 mL). The reaction atmosphere was flushed with argon gas and then, palladium on carbon (Pd/C, 10 wt.% loading) (35 mg, 0.33 mmol) was added in one‐portion. Then, hydrogen gas was constantly bubbled through the reaction, while stirred vigorously, for 2 h. Upon completion as assessed by LCMS, the reaction was filtered through cotton and washed thoroughly with methanol. The solvents were evaporated in‐vacuo to yield crude aniline intermediate, which was used without further purification. The intermediate was then re‐constituted in anhydrous DMF (1 mL), followed by the addition of compound 5 (20 mg, 0.034 mmol, 1 eq.), propanephosphonic acid anhydride (T3P) (50 wt.% in DMF) (200 µL, 0.34 mmol, 10 eq.) and TEA (47 µL, 0.34 mmol, 10 eq.). The reaction was allowed to stir overnight. The reaction was purified by RP‐HPLC (solvent system 2 was used, see Purification section) and lyophilized to afford the final product as a pink powder (Yield: 6 mg, 16%). After characterization, a stock of FAP‐FLASH550 was prepared as a 1 mm solution in DMSO. ^1^H NMR (400 MHz, CD_3_OD, ppm): δ = 8.73 (d, J = 4.5 Hz, 1H), 8.53 (s, 3H), 7.97–7.90 (m, 2H), 7.84–7.75 (m, 2H), 7.61 (d, J = 8.7 Hz, 2H), 7.55 (d, J = 4.5 Hz, 1H), 7.49–7.39 (m, 2H), 7.25 (d, J = 8.8 Hz, 2H), 6.78 (s, 2H), 6.52 (s, 1H), 5.11 (dd, J = 9.3, 3.2 Hz, 1H), 4.60 (s, 4H), 4.33–4.20 (m, 4H), 3.14 (s, 2H), 2.80–2.38 (m, 9H), 2.32–2.20 (m, 2H), 2.07 (d, J = 7.6 Hz, 2H), 1.72 (s, 4H). MS (ESI): m/z calcd for C_61_H_61_F_2_N_12_O_7_^+^: 1111.47 [M]^+^, found: 1111.80. Fluorescence (DMSO/PBS, nm): λ_ex_ = 550 nm; λ_em_ = 570 nm.

#### 4.4.6 Fragment 1

To a solution of compound 2 (10 mg, 0.024 mmol, 1 eq.) dissolved in anhydrous dimethylformamide (DMF) (0.5 mL), 4‐(4,6‐Dimethoxy‐1,3,5‐triazin‐2‐yl)‐4‐methylmorpholinium chloride (DMTMM) (27 mg, 0.096 mmol, 4 eq.) and triethylamine (TEA) (6.7 µL, 0.048 mmol, 2 eq.) were added. The reaction was allowed to stir for 1 h. Then, methylamine (2 M in THF) (48 µL, 0.096 mmol, 4 eq.) was added drop‐wise and the reaction was allowed to stir for another 2 h. The reaction was immediately purified by RP‐HPLC (solvent system 1 was used, see Purification section) and lyophilized to yield the product as a pink powder (Yield: 5 mg, 50%). ^1^H NMR (400 MHz, [D_6_]DMSO, ppm): δ 7.67 (d, J = 6.6, 1H), 7.40 (m, 2H), 6.91 (d, J = 6.5, 1H), 6.13 (s, 2H), 5.96 (s, 2H), 5.88 (s, 2H), 3.05 (s, 4H), 2.42 (s, 3H), 2.40–2.24 (m, 2H), 1.61 (m, 4H). MS (ESI): m/z calcd for C_27_H_25_N_3_O_2_: 424.20 [M]^+^, found: 424.25.

### 4.5 Fluorescence Spectra

#### 4.5.1 Fluorescence Excitation and Emission Spectra

The fluorescence excitation and emission spectra of FAP‐FLASH550 was measured using a QuantaMaster 400 fluorimeter (PTI, New Jersey, USA). A stock of probe (1 mm) in DMSO was diluted (1:1000, 2 mL) to a final concentration of 1 µm in 50% DMSO/PBS and transferred to a fluorimeter cuvette (Sigma–Aldrich, Cat. C0918‐100EA) for analysis. Excitation (λ_ex_, 400–570 nm; λ_em_, 580 nm) and emission (λ_ex_, 550 nm; λ_em_, 565–650 nm) spectra were measured in 1 nm steps using a slit width of 2 nm.

#### 4.5.2 Spectroscopic Study

A solution of FAP‐FLASH550 (final concentration, 50 µm; total volume, 200 µL) in Phosphate‐Citrate buffer (pH 8) was irradiated with a UV LED Spot Light (365 nm, 2.05 mW/cm^2^, Advanced Illumination Model No.: SL223S‐365IC) for 1 min. As a control, a solution of FAP‐FLASH550 (final concentration, 50 µm; total volume, 200 µL) in Phosphate‐Citrate buffer (pH, 8) was prepared, which was not exposed to UV light. Then, 100 µL of each solution was transferred to either a black, flat‐bottom 96‐well plate (Greiner Bio‐One, Cat No.: 655086). The fluorescence emission spectrum (λ_ex_, 550 nm; λ_em_, 565–650 nm) was obtained for each well using a monochromator‐based multi‐well plate reader (Tecan, Spark Multimode Micro‐plate Reader).

#### 4.5.3 Fluorescence Quenching Rate

A solution of FAP‐FLASH550 (final concentration, 5 µm; total volume, 100 µL) in PBS (pH, 7.4) or Phosphate‐Citrate buffer (pH, 8) in a 0.5 mL Eppendorf tube was irradiated with a UV light (365 nm, 2.05 mW/cm^2^) or green light (525 ± 36 nm) for various time‐points (i.e., 0, 5, 10, 20, 30, 40, 50, 60 s). After irradiation, 50 µL of each solution, followed by 50 µL of DMSO to ensure complete solubilization, was pipetted into a black, flat‐bottom 96‐well plate (Greiner Bio‐One, Cat No.: 655086). The fluorescence intensity (λ_ex_, 550 nm; λ_em_, 570 nm) was measured for each well using a monochromator‐based multi‐well plate reader (Tecan, Spark Multimode Micro‐plate Reader) and plotted against time (in seconds) using GraphPad Prism. The curve was fit using a non‐linear regression (one‐phase decay).

#### 4.5.4 Stability Study

A solution of FAP‐FLASH550 (final concentration, 50 µm; total volume, 100 µL) in various buffers: Phosphate‐Citrate buffer (pH, 8), PBS (pH, 7.4), or PBS (pH, 6.8) was transferred to a black, flat‐bottom 96‐well plate in triplicate and kept at room temperature. The fluorescence intensity (λ_ex_, 550 nm; λ_em_, 570 nm) was measured using a plate reader at several time‐points (0, 5, 30, 60, and 240 min). Then, the fluorescence intensity was plotted against time (in minutes) using GraphPad Prism.

### 4.6 FAP Inhibition Assay

The inhibitory effect of FAP‐FLASH550 was measured using the fluorogenic FAP substrate Z‐Gly‐Pro‐AMC (MedChemExpress, Cat. HYD1670) following a previously reported protocol [7].

### 4.7 Cell Culture

4T1 cells (Purchased 08/2018, RRID: CVCL_0125) and NIH/3T3 cells (Purchased 04/2011, RRID: CVCL_0594) were purchased from the American Type Culture Collection (ATCC). Cells are routinely screened for mycoplasma and were confirmed to be negative before all experiments. Cells were plated and cultured in Dulbecco's Modified Eagle Medium (DMEM) (Corning) supplemented with 10% Fetal Bovine Serum (Corning) and 1% Penicillin‐Streptomycin (Corning) at 37°C and 5% CO_2_. Cells were passaged with 0.05% trypsin‐EDTA (Corning). 4T1 cells were transfected with Venus GFP as previously described [22].

### 4.8 Cell Toxicity Assay

Twenty‐four hours before treatment, NIH/3T3 cells (5,000 per well) were plated in a 96‐well plate. On the day of treatment, a 1:5 serial dilution of FAP‐FLASH550 was prepared in complete medium spanning concentrations from 0.008 to 5 µm (total volume, 100 µL) and applied to cells for 2 min. Each probe concentration was applied in triplicate. Following treatment, the cells were washed with media before incubating in fresh medium for 72 h. After incubation, the cells were treated with a 10X dilution of Presto Blue (Thermo Fisher Scientific) and incubated for 60 min at 37°C. The fluorescence intensity (λ_ex_, 560 nm; λ_em_, 590 nm) was measured using a multi‐mode microplate reader.

### 4.9 Mouse Models

All animals were obtained from Jackson Laboratory (Stock # 000651) and housed under specific pathogen‐free conditions at Massachusetts General Hospital. Experiments were approved by the MGH Institutional Animal Care and Use Committee (IACUC) and were performed according to MGH IACUCAC protocol 2013N000157. All mice were provided ad libitum access to food and water and a standard 12‐h light/dark cycle. All experiments were performed under isoflurane gas anesthesia. 10‐week‐old female BALB/C mice were utilized for all experiments. A total of *n* = 20 mice were used. This included *n* = 10 mice for ex vivo tumor imaging and *n* = 10 mice for fluorescence‐guided surgery.

#### 4.9.1 Tumor Model

4T1‐Venus GFP tumor cells (5 × 10^5^) in 100 µL of serum‐free DMEM were injected into the fourth right mammary fat pad of female BALB/C mice and allowed to grow for 7 days before excision.

### 4.10 Ex Vivo Tissue Staining

Excised 4T1 Venus‐GFP tumor and adipose tissues were embedded in agarose (4% w/v in H_2_O) and cut into 300 µm sections using a vibrating microtome. Tissue sections were fully submerged in FAP‐FLASH550 (5 µm) solutions diluted in PBS for 2‐min before submerging in PBS for a 5‐min wash. Following imaging, tumor‐to‐background tissue ratios (TBRs) were calculated from the average fluorescence intensity of each tissue type.

### 4.11 Fluorescence Guided Surgery in Live Tumor‐Bearing Mice

Fluorescence guided surgery was performed using a custom‐built intraoperative imaging system using an epifluorescence intraoperative microscope with a 0.65–5 variable objective. Briefly, images were collected in white light, GFP (λ_ex_, 455 ± 25 nm; λ_em_, 543 ± 30 nm) and TRITC (λ_ex_, 525 ± 36 nm; λ_em_, 585 ± 14 nm) channels. On the day of imaging, mice bearing orthotopic 4T1‐Venus GFP mammary tumors were anesthetized and positioned under the imaging system. The tumor was exposed by making three cuts to the abdominal skin: A midline cut followed by two transverse cuts above and below the tumor. Once exposed, the operative field was treated with 3% calcium chloride before staining with ESOP (5 µm) diluted in alginate (0.5% w/v in H_2_O) for 2 min. After staining, the field was rinsed with EDTA (3% in PBS) for 1 min, followed by a solution of PBS for 4 min. All solutions were sprayed onto the operative field using a commercial spray device (MADomizer bottle, Teleflex). Fluorescence imaging was performed in each channel. Then, the operating field was irradiated with a UV LED Spot Light (365 nm, 2.05 mW/cm^2^, Advanced Illumination Model No.: SL223S‐365IC) for 2‐min and then, re‐imaged. Afterward, the operating field was re‐stained and re‐imaged.

### 4.12 Histology

4T1 orthotopic mammary tumors were harvested and fixed in 4% paraformaldahyde solution. The tumors were paraffin‐embedded and sectioned at 5 µm. The sections were then deparaffinized and rehydrated before staining. H&E staining was performed according to manufacturer's protocol (ab245880, Abcam). All the slides were scanned using the NanoZoomer 2.0RS scanner (Hamamatsu) for analysis.

### 4.13 Image Analysis and Quantification

All image visualization and quantification was performed with Fiji (ImageJ, 2.14.0/1.54F). Images were automatically windowed and leveled to maximize contrast and false‐colored to differentiate channels.

### 4.14 Statistics

All statistical data analyses were performed using GraphPad Prism 9 software and results are expressed as mean ± standard deviation. For normally‐distributed datasets, we used two‐tailed Student's *t*‐test and one‐way ANOVA followed by Bonferroni's multiple comparison test. When variables were not normally distributed, we performed non‐parametric Mann–Whitney or Kuskal–Wallis tests. *p*‐values > 0.05 were considered not significant (n.s.), *p*‐values < 0.05 were considered significant.

## Author Contributions

Conceptualization: ZR, RJDW, RW. Data Curation: ZR, RJDW. Formal Analysis: ZR. Methodology: ZR, RJDW, RW. Validation: RW. Supervision: RW. Visualization: ZR, RJDW, RW. Writing – original Draft: ZR, RW. Writing – review and Editing: RW and all Coauthors. Funding Acquisition: RW. Project Administration: RW. Resources: RW.

## Conflicts of Interest

The authors declare no conflicts of interest.

## Supporting information




**Supporting File**: advs74999‐sup‐0001‐SuppMat.docx.

## Data Availability

The data that support the findings of this study are available in the supplementary material of this article.
