# Supplementary material for: Erasable FAP Targeted Spray‐On Probe for Fluorescence‐Guided Surgery
Source: Adv Sci (Weinh). 2026 Mar 24;13(32):e23986. doi: 10.1002/advs.202523986 (PMC13252619; doi:10.1002/advs.202523986)
Supplement: Supplementary file 1 — Supporting File: advs74999‐sup‐0001‐SuppMat.docx. [file ADVS-13-e23986-s001.docx]

Erasable FAP targeted spray-on probe for fluorescence-guided surgery

Zachary Rabinowitz^1†^, Riley J. Deutsch-Williams^1†^, Ralph Weissleder^1,2,^*

^†^These authors contributed equally

^1^ Center for Systems Biology, Massachusetts General Hospital, 185 Cambridge St, CPZN 5206, Boston, MA 02114,

^2^ Department of Systems Biology, Harvard Medical School, 200 Longwood Ave, Boston, MA 02115

*R. Weissleder, MD, PhD

Center for Systems Biology

Massachusetts General Hospital

185 Cambridge St, CPZN 5206

Boston, MA, 02114

617-726-8226

[rweissleder@mgh.harvard.edu](mailto:weissleder@helix.mgh.harvard.edu)

Keywords: FAP, fibroblast, cancer, fluorescence, surgery, rhodamines

**Fig. S1: Difference between “Always-on” SOP and “Erasable” SOP.** **A.** “Always-on” SOPs can be sprayed onto the operating field and after a short stain-and-wash procedure (2 min stain then 5 min wash), highly sensitive tumor-to-background ratios (TBRs) could be achieved. However, repeated staining can lead to the accumulation of non-specific signal in normal adjacent tissue, thereby decreasing the tumor-to-b
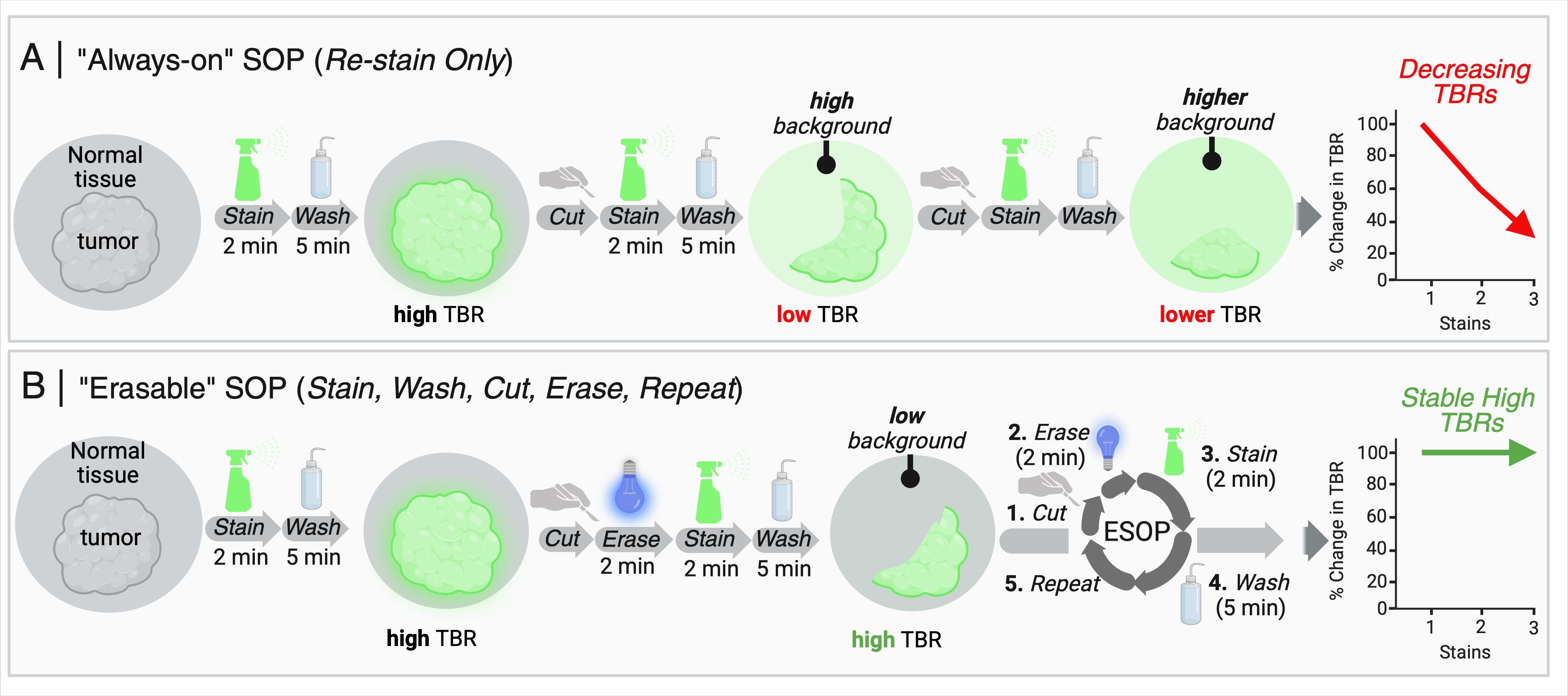
ackground ratio over multiple applications. **B.** To circumvent this problem, erasable probes (ESOPs) can theoretically lead to stable high TBRs using an erase and re-stain workflow. After topical application via spray, ESOPs could stain tumor margins and the fluorescent signal can then be erased using a brief UV light pulse to allow for repeated tissue staining and minimize build-up of non-specific signal.

**Fig. S2: Purity and mass spectrometry analysis of synthesized compounds via HPLC-MS.** HPLC traces of (**A.**) compound 2, (**B.**) compound 4, (**C.**) compound 5, (**D.**) compound 1, and (**E.**) fragment 1. Each compound eluted as a single sharp, narrow peak with a purity >95%. Mass spectra (ESI-MS) of (**F.**) compound 2, (**G.**) compound 4, (**H.**) compound 5, (**I.**) compound 1, and (**J.**) fragment 1. The observed masses were consistent with calculated values for each compound, confirming the successful generation of each compound.


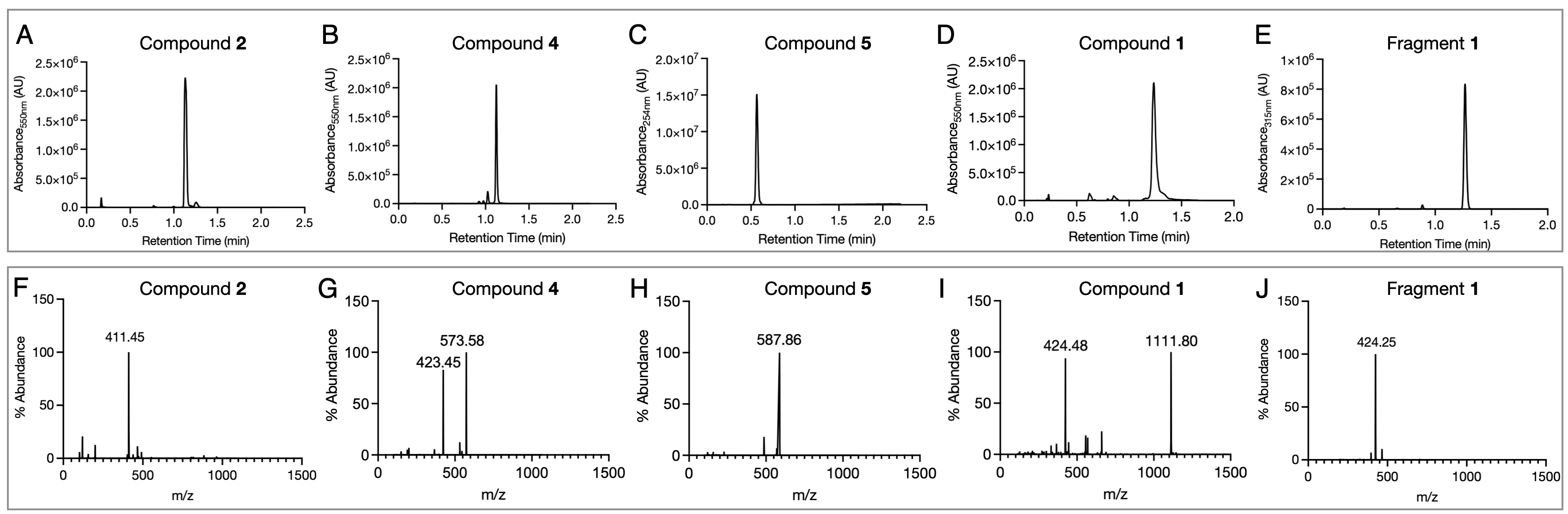


**
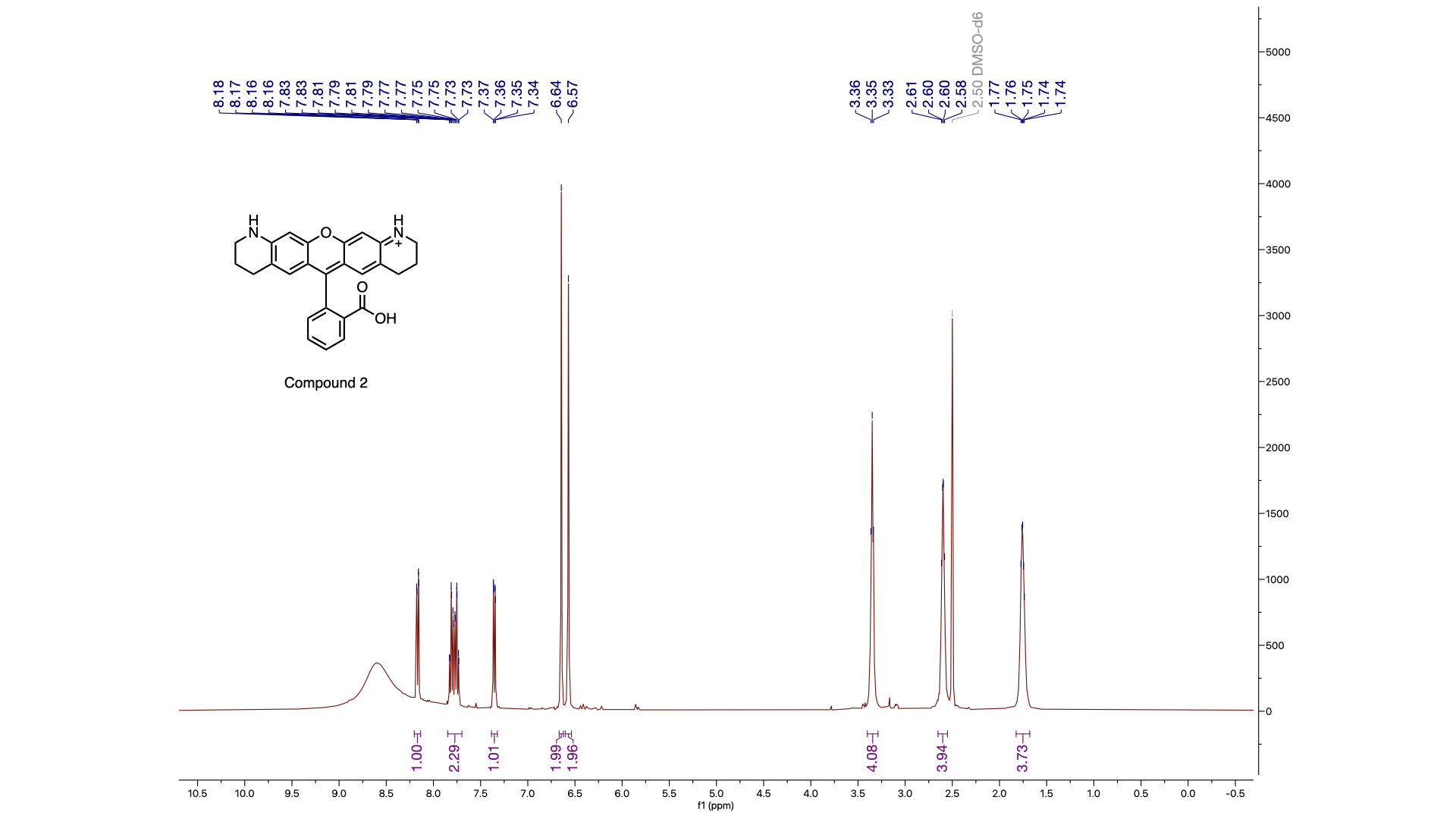
Fig. S3: ^1^H NMR of Compound 2 in DMSO-d6**

**Fig. S4: ^1^H NMR of Compound 3 in DMSO-d6**


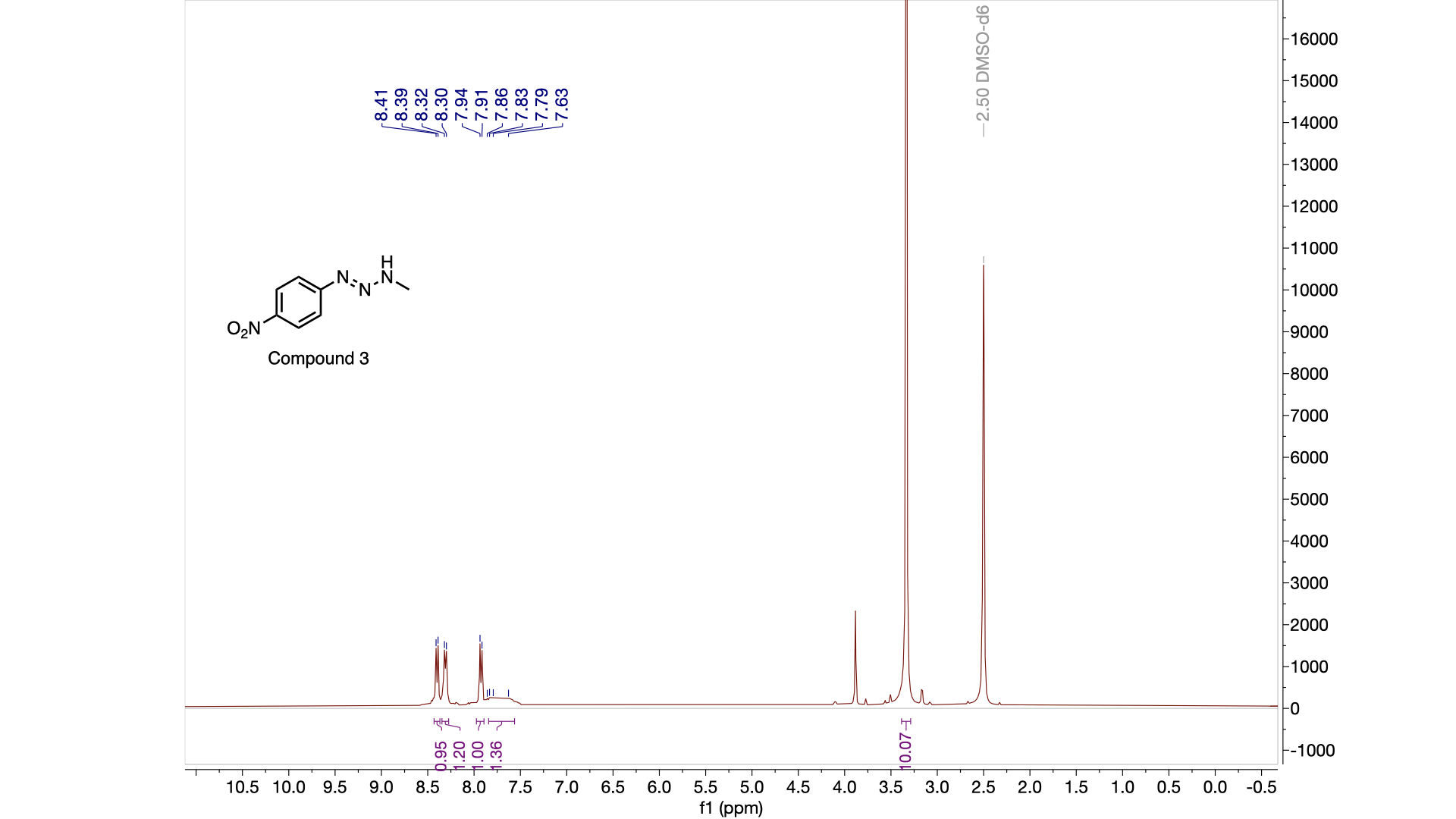


**Fig. S5: ^1^H NMR of Compound 4 in DMSO-d6.**


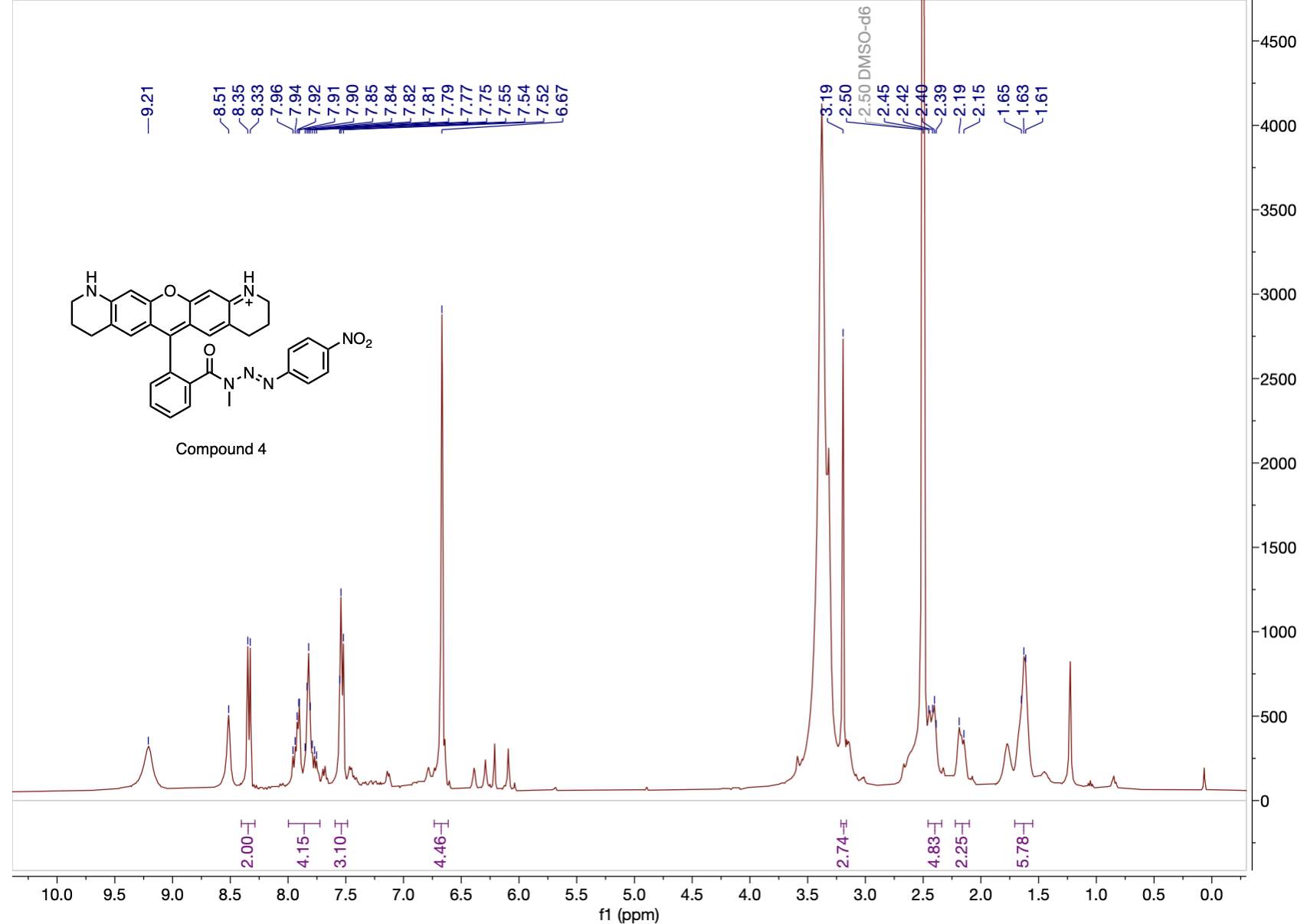


**Fig. S6: ^1^H NMR of Compound 5 in CD_3_OD.**


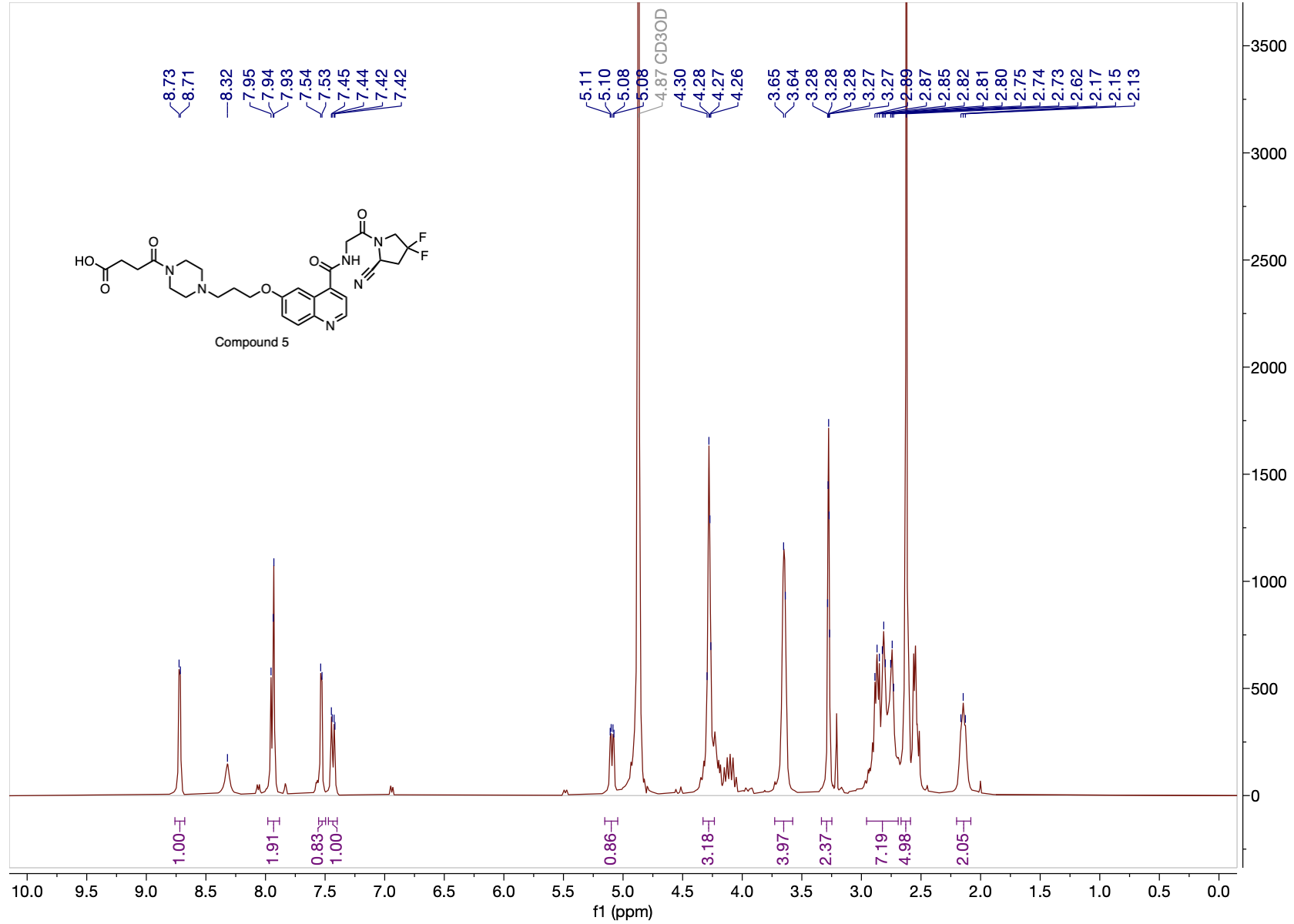


**Fig. S7: ^1^H NMR of Compound 1 (FAP-FLASH550) in CD_3_OD.**


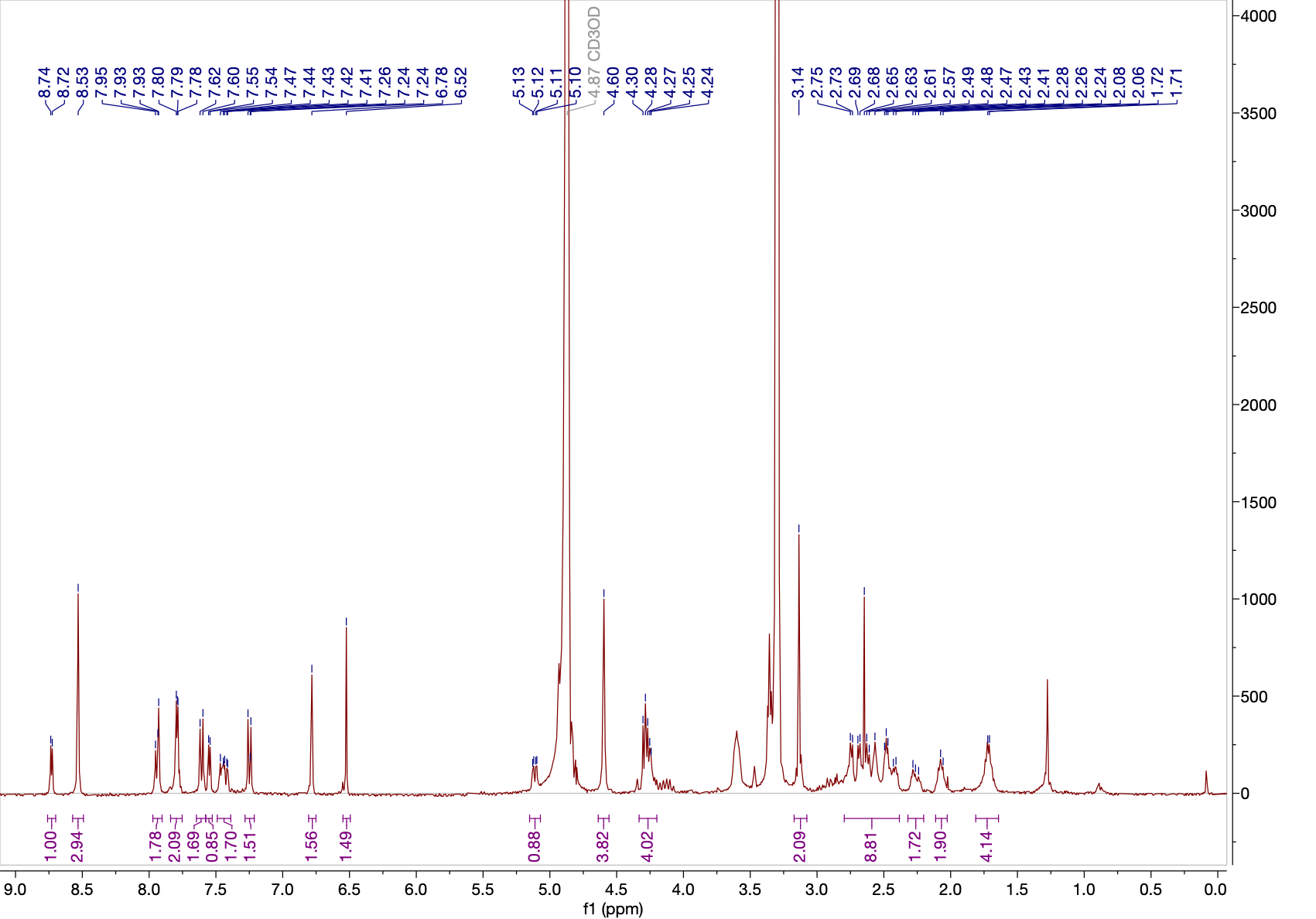

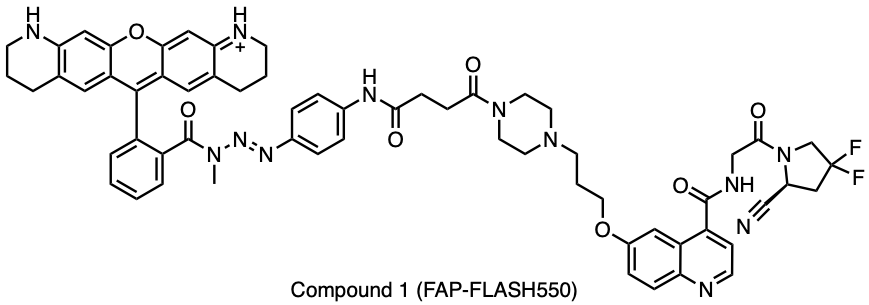


**Fig. S8: ^1^H NMR of Fragment 1 in DMSO-d6**

**
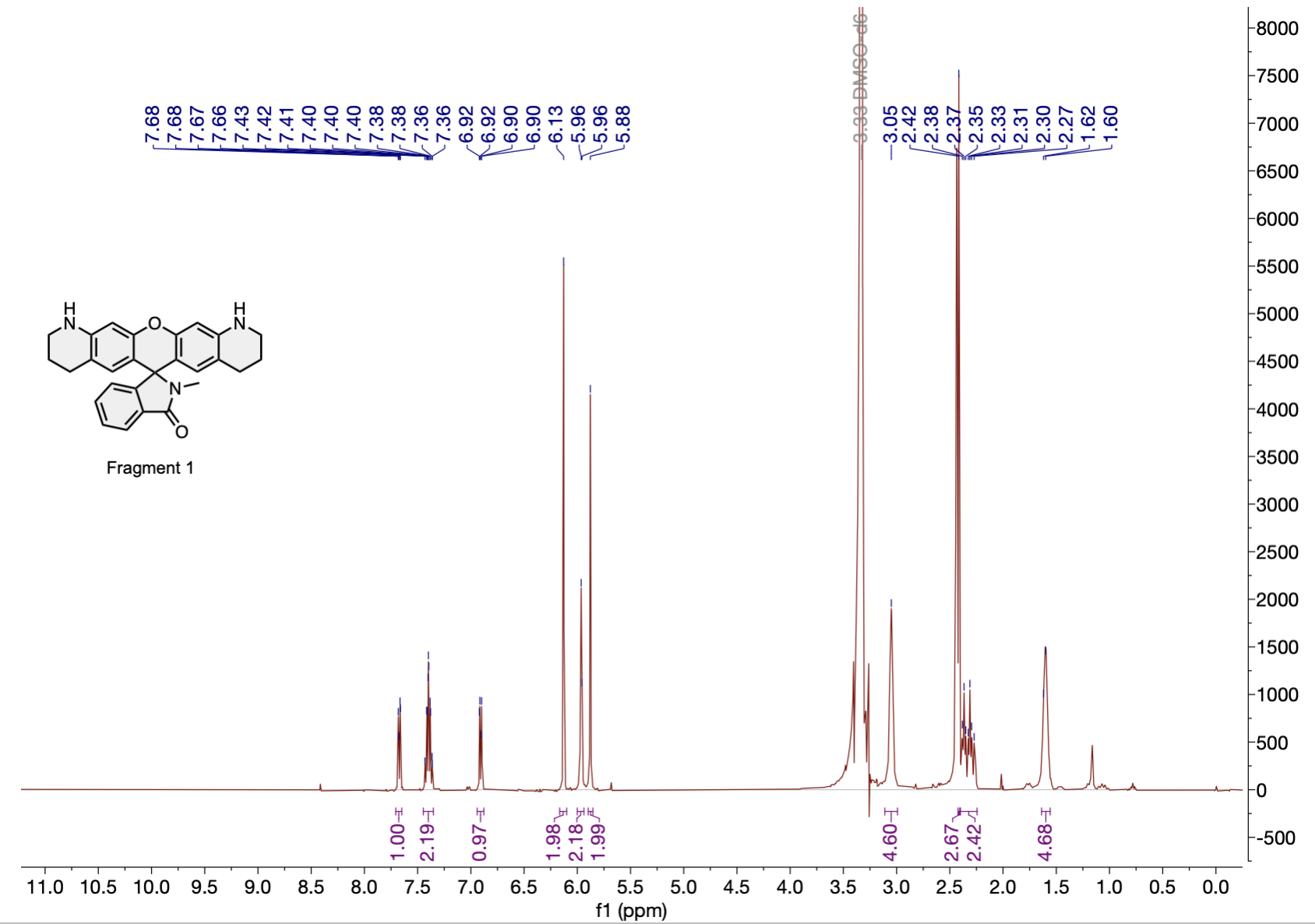
**

**Fig. S9: Fluorescence stability of FAP-FLASH550 in aqueous buffer.** **A.** Stability of FAP-FLASH550 (50 μM) in aqueous buffers at pH 6.8, 7.4, and 8.0, assessed by fluorescence intensity measurements (λ_ex_/λ_em_ = 550/570 nm). Fluorescence intensity remained stable over 4 h, indicating robust probe stability under aqueous conditions.**B.** No evidence of nonspecific hydrolysis of FAP-FLASH550 in aqueous buffers at room
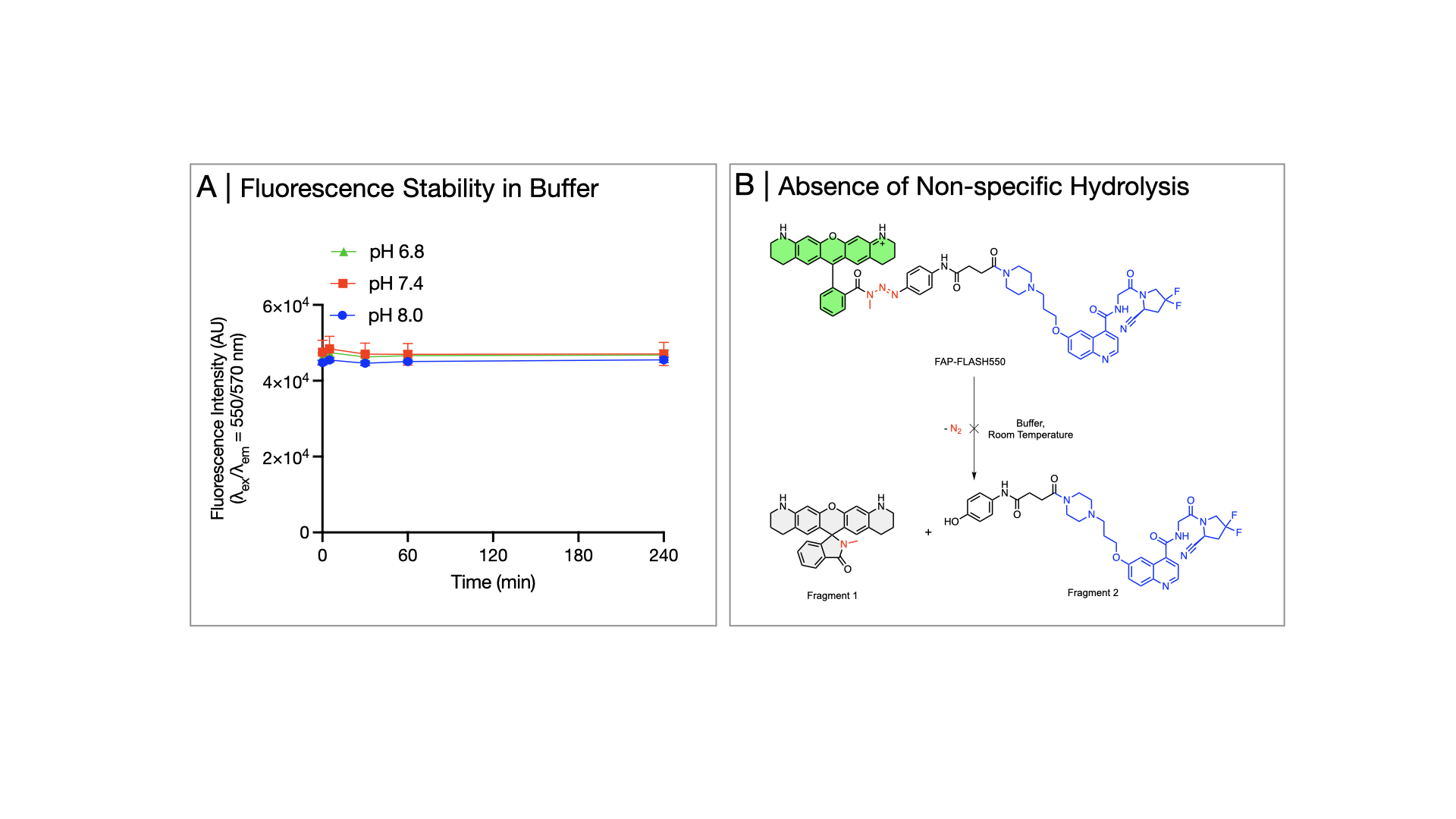
temperature, consistent with the fluorescence stability observed in A.

**Fig. S10: Comparing the fluorescence quenching of FAP-FLASH550 in either phosphate-buffered saline (PBS) (pH, 7.4) or phosphate-citrate buffer (pH, 8.0).** A solution of FAP-FLASH550 (5 µM) in either phosphate-buffered saline (pH, 7.4) or phosphate-citrate buffer (pH, 8.0) was exposed to a UV light pulse (365 nm) for various time-points (0-60 seconds) and the fluorescence intensity was measured at 570 nm (λ_ex_, 550 nm). With a pulse of UV light (365 nm), the reaction proceeded extremely fast at both pH 7.4 and pH 8, with an average fluorescence half-life of 1.4 and 1.9 seconds, respectively. This confirms that the fluorescence quenching reaction can also occur at physiological pH (pH, 7.4).


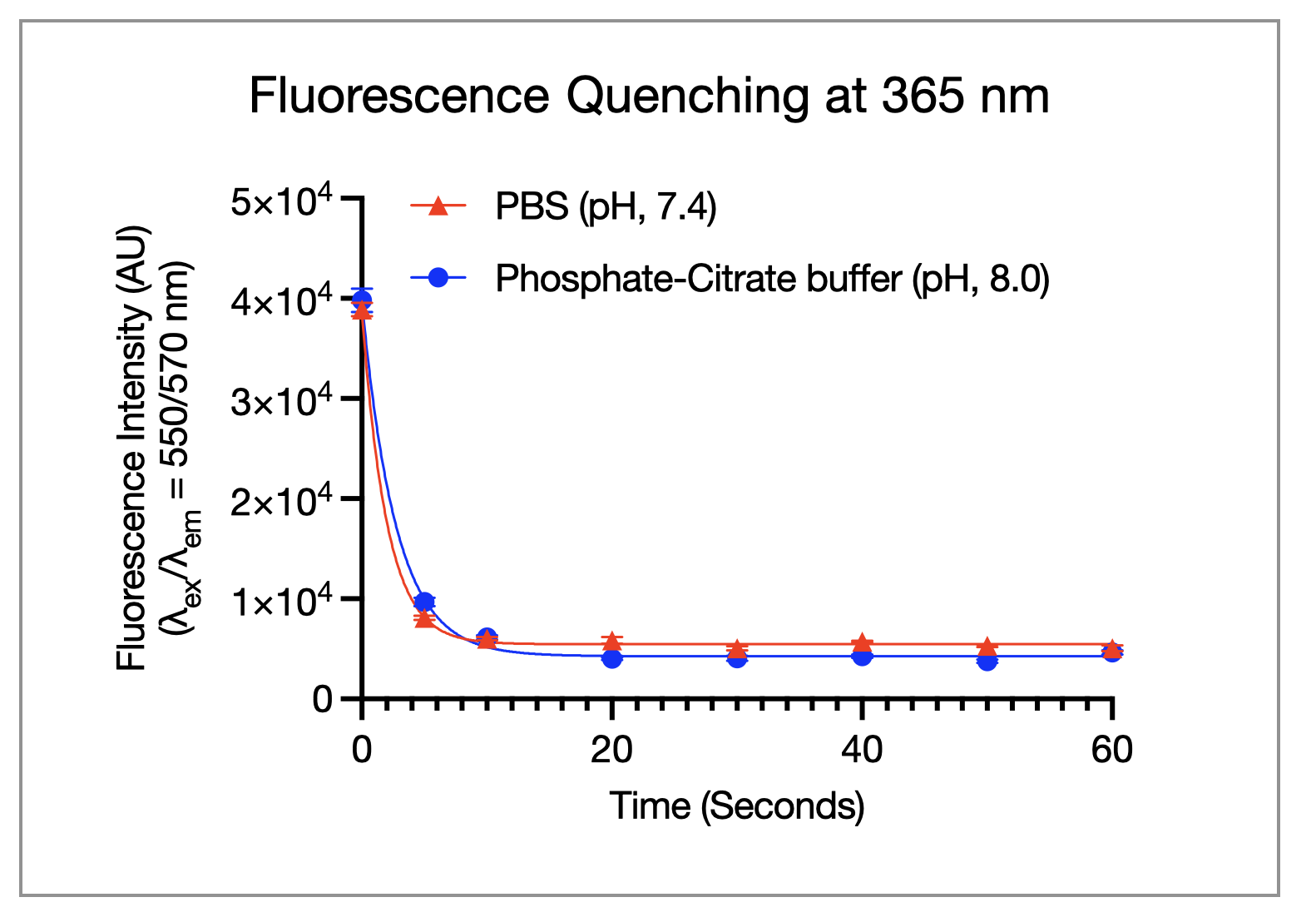


**Fig. S11: pH dependence of fluorescence emission of Fragment 1. A.** Two-step, one-pot synthesis of Fragment 1 starting from rhodamine compound 2. **B.** The pH-dependent fluorescence emission of Fragment 1. The fluorescence intensity (λex/λem = 550/570 nm) of Fragment 1 (5 μM) was measured in buffers ranging from pH 3 to 9 (n = 3) and plotted as a function of buffer pH. Fragment 1 is non-fluorescent at pH values above 7 (shaded in gray), while fluorescence increases under acidic conditions (shaded in light green). Because the pH of the surgical field is typically ~7.4–8.0, the fluorophore is expected to remain in a non-fluorescent state under surgical conditions. **C.** pH-dependent spirocyclization of Fragment 1. Under acidic conditions, the equilibrium favors the open, fluorescent form, whereas under basic conditions it shifts toward the closed, colorless spirolactam form, consistent with the fluorescence behavior observed
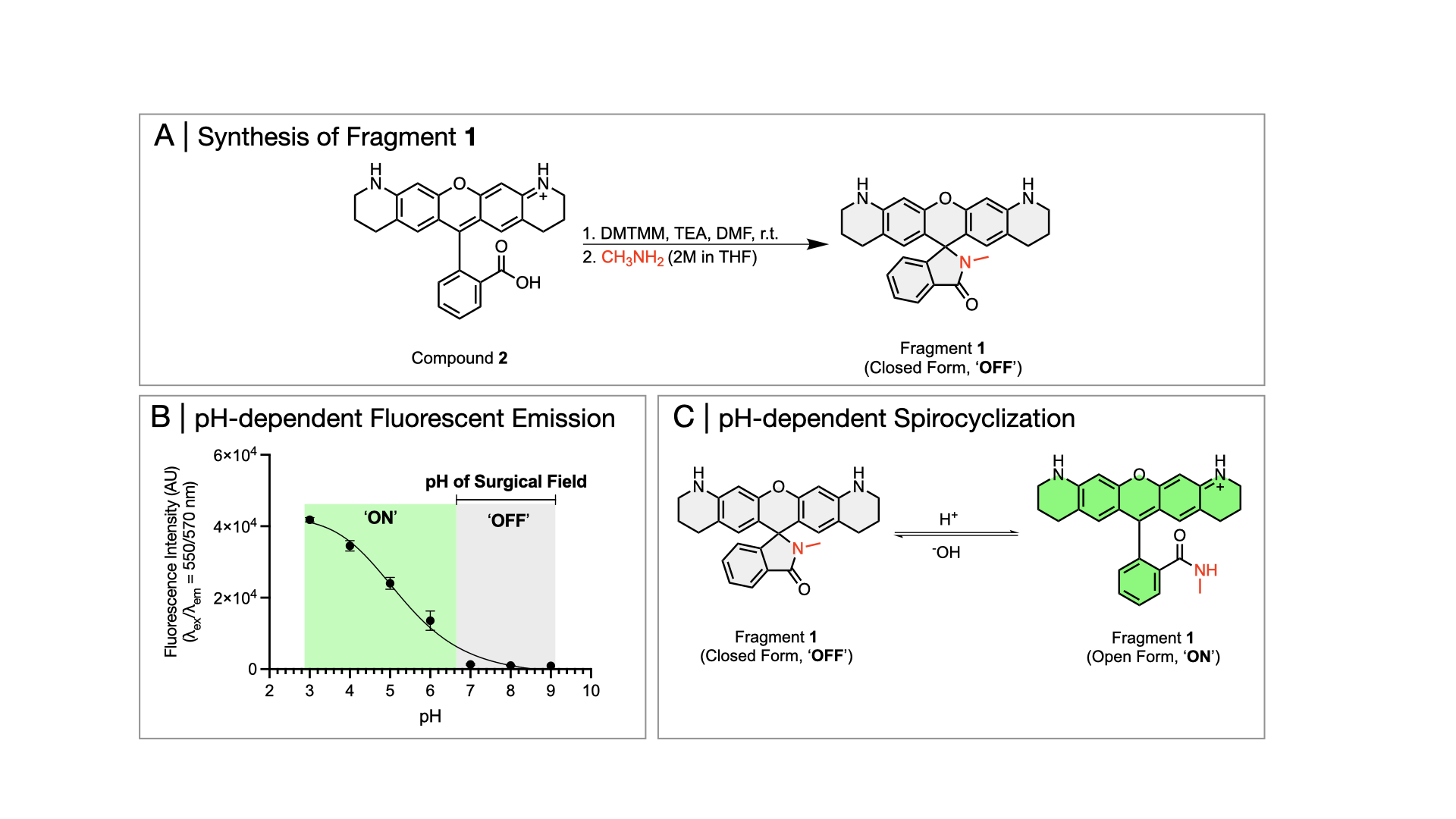
in B.

**Fig. S12: Binding affinity of FAP-FLASH550 to murine FAP (mFAP) using the fluorogenic Z-Gly-Pro-AMC assay.** FAP-FLASH550 exhibited remarkable single-digit nanomolar binding affinity (IC_50_ = 3.00 nM) to mFAP.
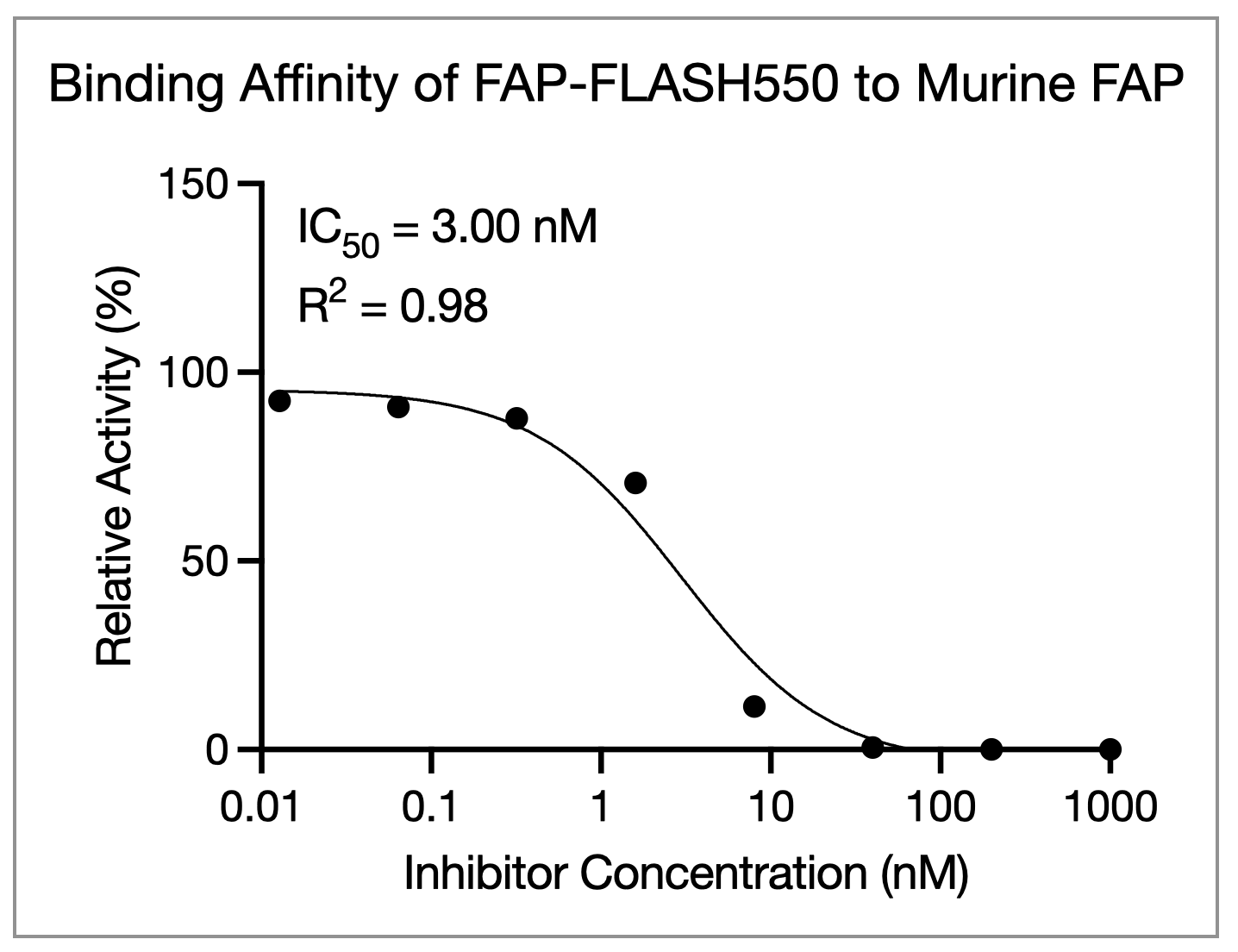


**Fig. S13: In vitro concentration-dependent toxicity of FAP-FLASH550.** Cells were treated with FAP-FLASH550 at various concentrations (0.008-5 μM) in complete medium for 2 min before incubating for 72 hours in fresh media. Red vertical line
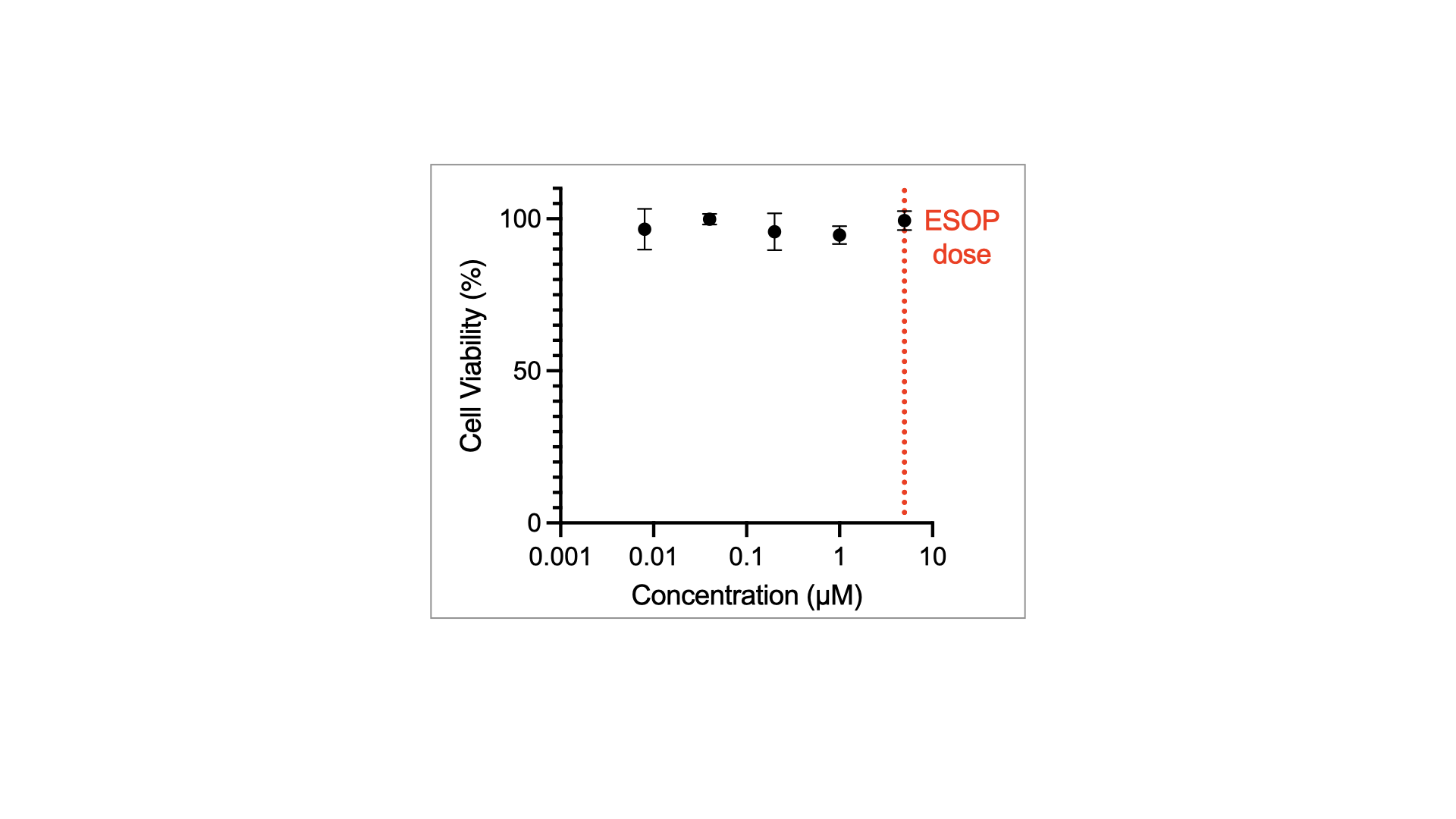
indicates the concentration used for ESOP imaging.

**Fig. S14: Optimizing the staining protocol of FAP-FLASH550. A**. Representative images of ex vivo 4T1 tumor sections (300 μm) and gonadal fat pad sections (300 μm) stained with various concentrations (0.5, 1, 5, 10 μM) at variable staining times (2, 5, 10 min), followed by a 5-minute PBS wash. **B**. Tumor-to-background ratios (TBRs) as function of probe concentration and stain time, as measured from **A**. Red arrow points to the optimized condition: 2-minute stain at a probe concentration of 5 μM.
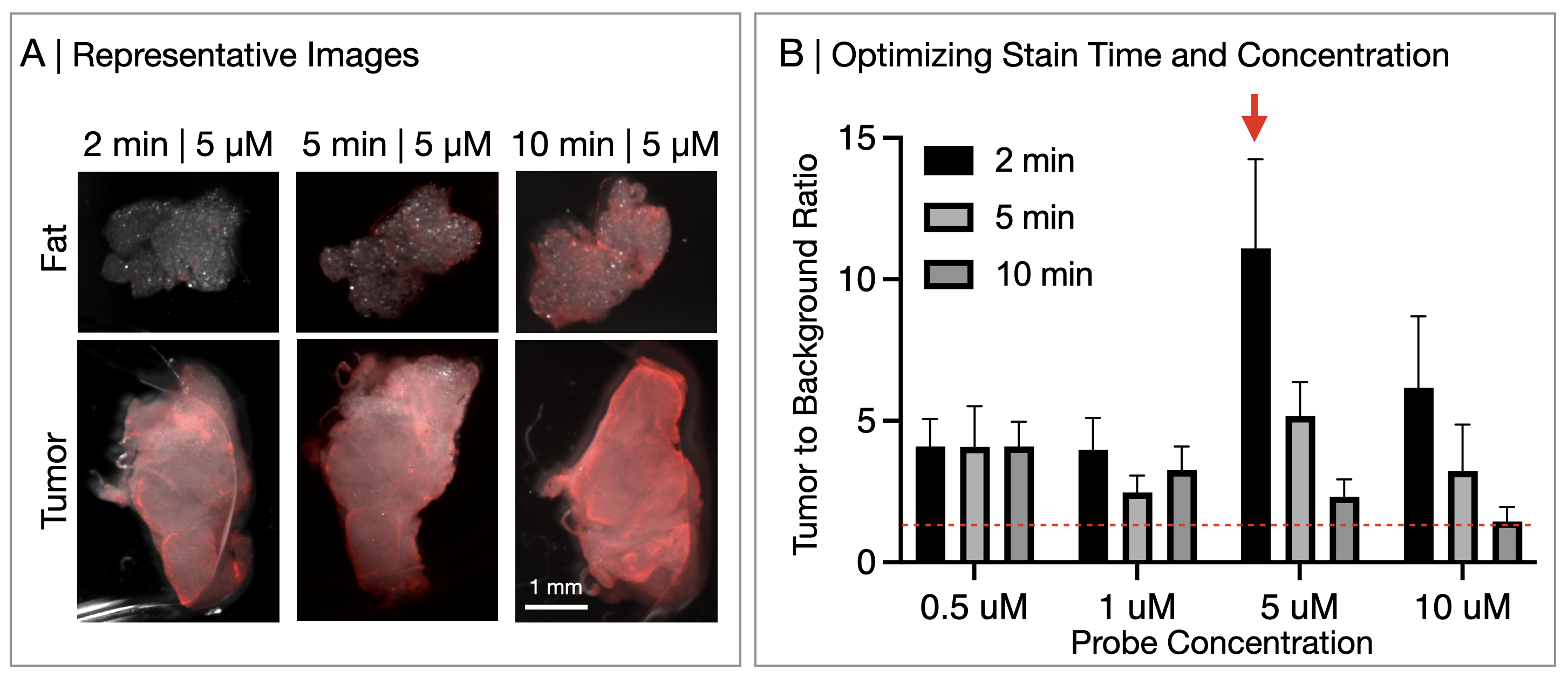


**Fig. S15: Images of tumor tissue (n = 3) and fat tissue (n = 3) repeatedly stained with FAP-FLASH550 using two different protocols. A.** "Erase and re-stain” protocol. Tissue slices (300 um) of 4T1 tumors (n = 3) or fat (n =3) were stained with FAP-FLASH550 (5 uM) for 2-minutes, followed by a 5-minute PBS wash and imaged using an epifluorescence microscope. Then, the fluorescent signals on tissues were erased via a 2-minute pulse of UV light (365 min), imaged, and then, re-stained. This process was repeated three times. **B.** “Re-stain only” protocol. Similarly, 4T1 tumor or fat slices were stained with FAP-FLASH550 (5 uM) for 2-minutes, followed by a 5-minute PBS wash. After imaging, the tissue sections were thrice re-stained following the same staining protocol. Imaging channels: Bright field (gray); FAP-FLASH550 (red) (λ_ex_, 525±36 nm; λ_em_, 585±14 nm). Fat tissue was used as non-tumor tissue control.
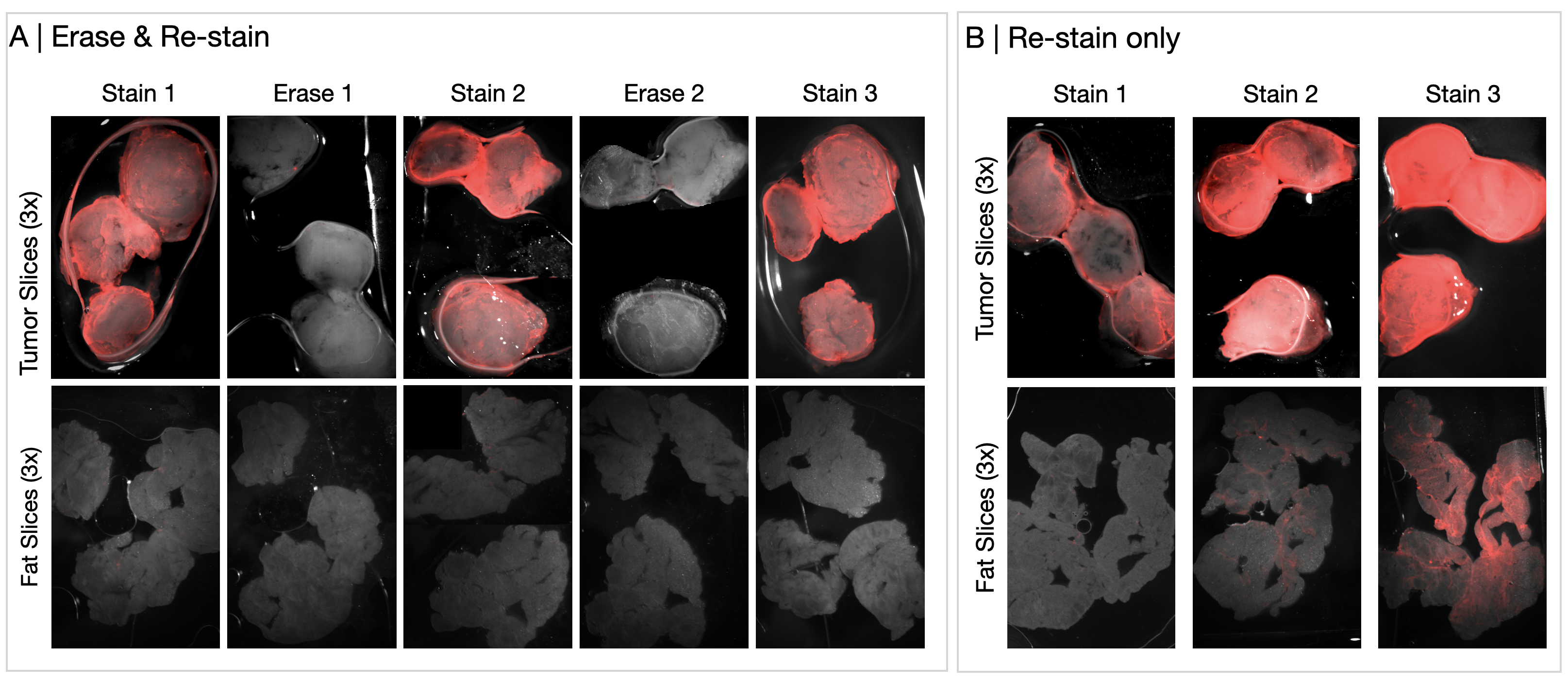


**Fig. S16: Representative image of ex vivo tumor tissue with adjacent mammary tissue after being administered intravenously with FTF-Cy5.** Briefly, 4T1 Venus-GFP mice bearing mice were injected with FTF-Cy5 (200 ug) intravenously via tail vein injection. After 1 hr, 4T1 Venus-GFP tumors with surrounding normal mammary tissue were resected and imaged using an epifluorescence microscope. Images were collected in white light (gray), GFP (green) (λ_ex,_ 455±25 nm; λ_em_, 543±30 nm) and Cy5 (red) (λ_ex_, 605±26 nm; λ_em_: 675±35) channels.


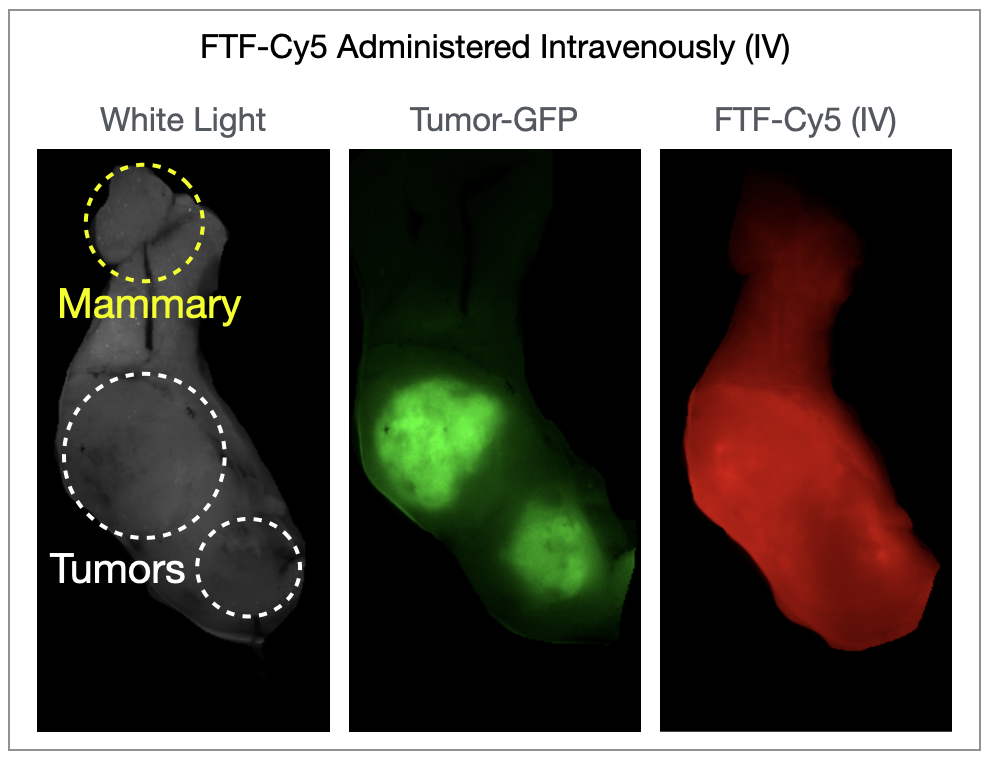


**Fig. S17: Tumor-to-background ratios (TBRs) for in vivo tumor staining.** Quantification of TBRs for 4T1 mammary tumors after staining with ESOP, after UV-mediated erasure, and after re-staining. Representation images shown in Fig. 4B. Error bars represent standard deviation.


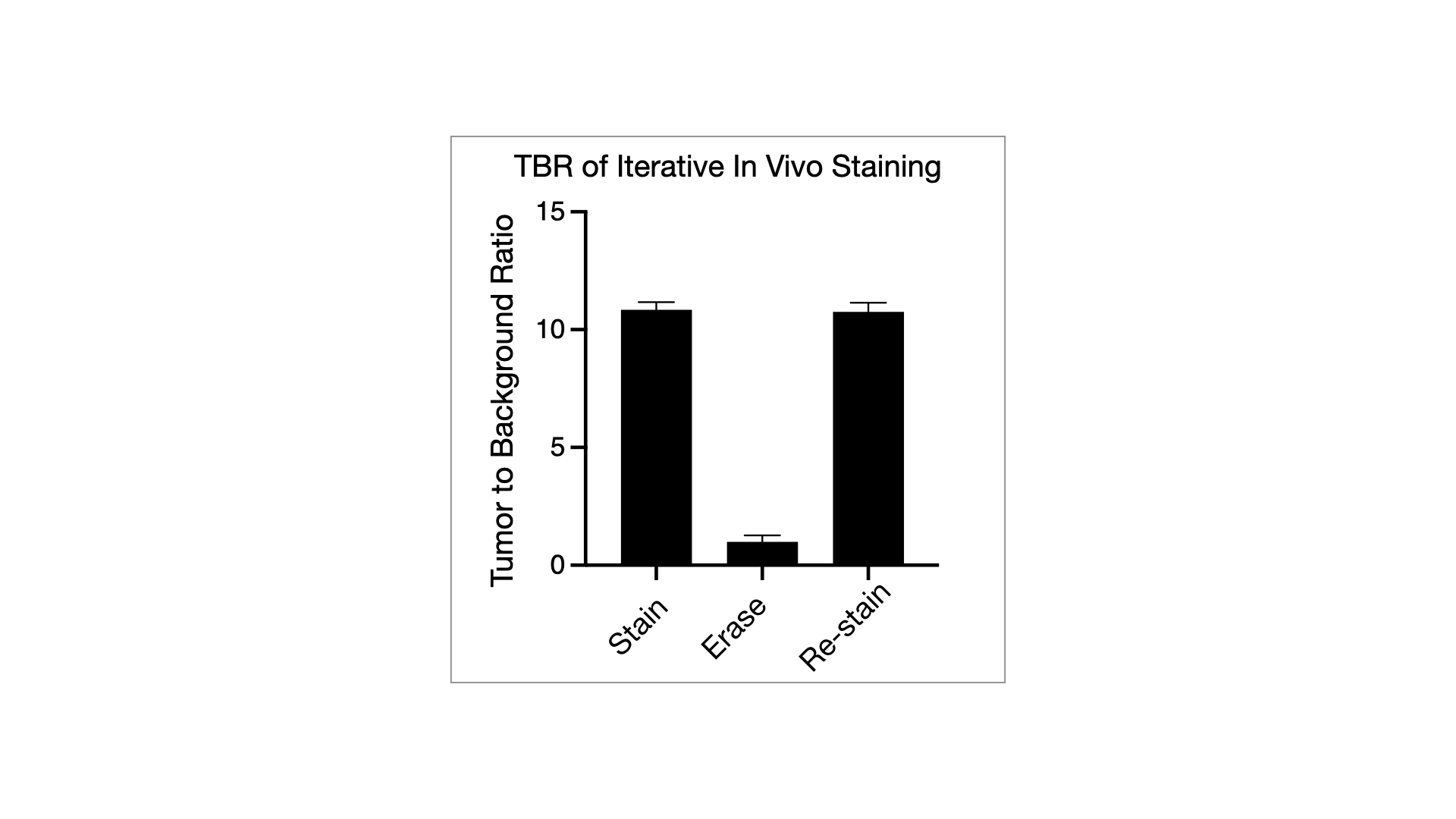


**Fig. S18: In vivo UV-mediated fluorescence erasing kinetics of FAP-FLASH550.** **A.** Representative surgical field with FAP-FLASH550 fluorescence (yellow) overlaid on a bright-field image (gray). **B.** Time course images of ESOP signal change during UV-mediated fluorescence quenching. Tissue was exposed to 10 second intervals of UV light between image capture.


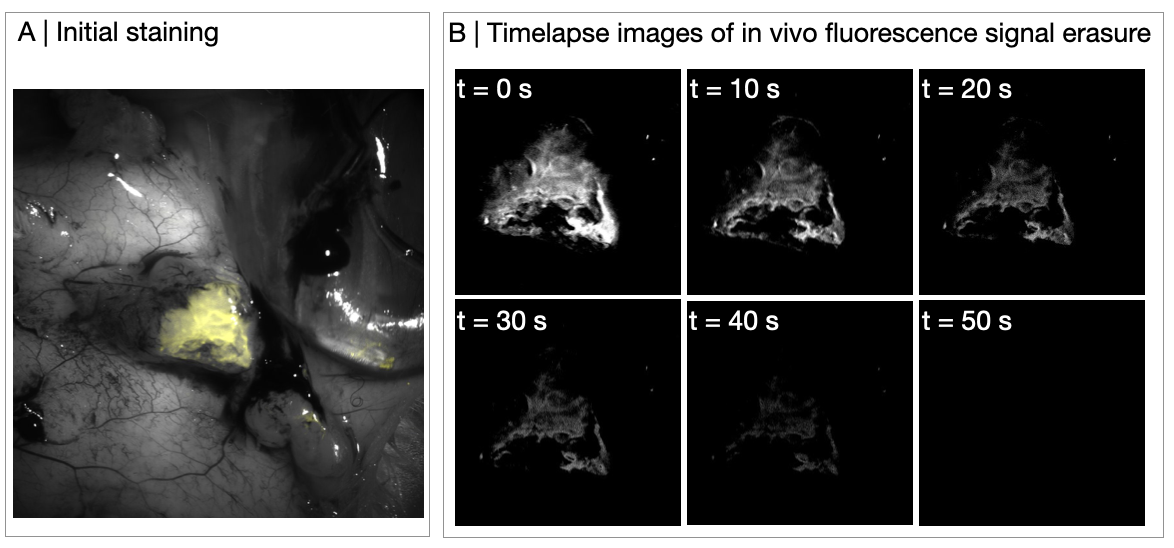


**Fig. S19:** **Comparison of FAP-FLASH550 signal distribution to histology.** 4T1 mammary tumor was stained with FAP-FLASH550, before being resected, fixed, and paraffin embedded for histology. Histological sections were stained with hematoxylin and eosin (H&E). Red regions correspond to mammary gland and cyan regions identify tumor regions in both images. Note, the relatively smaller region of adipose the H&E
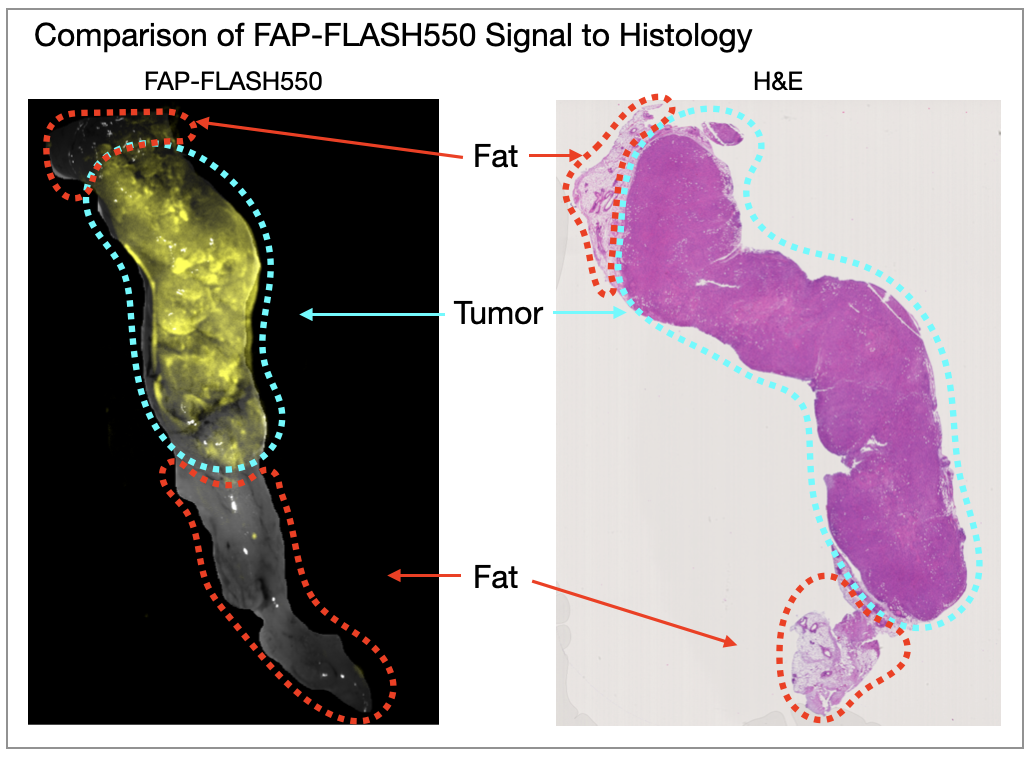
section due to tissue deformation throughout processing steps.

**Figure S20: Validation turn-off stability at various pH values. A.** Experimental Workflow. Briefly, tumors were stained, imaged, and then quenched with a 2 min UV light pulse (365 nm, 2.05 mW/cm^2^). Subsequently, samples were incubated at pH 8 for two minutes and then re-imaged. Following this, the same sample was incubated at pH 6.5 for 2 minutes and imaged and finally incubated at pH 4.5 before a final round of imaging. This was done to assure that there is no residual fluorescence emission of Fragment 1 **B.** Quantification of fluorescence signal after each condition. Error bars acquired from the intensities of 3 FOVs. **C.** Representative images. FAP-FLASH550 signal is shown in red and tumor GFP-Venus is shown in green.


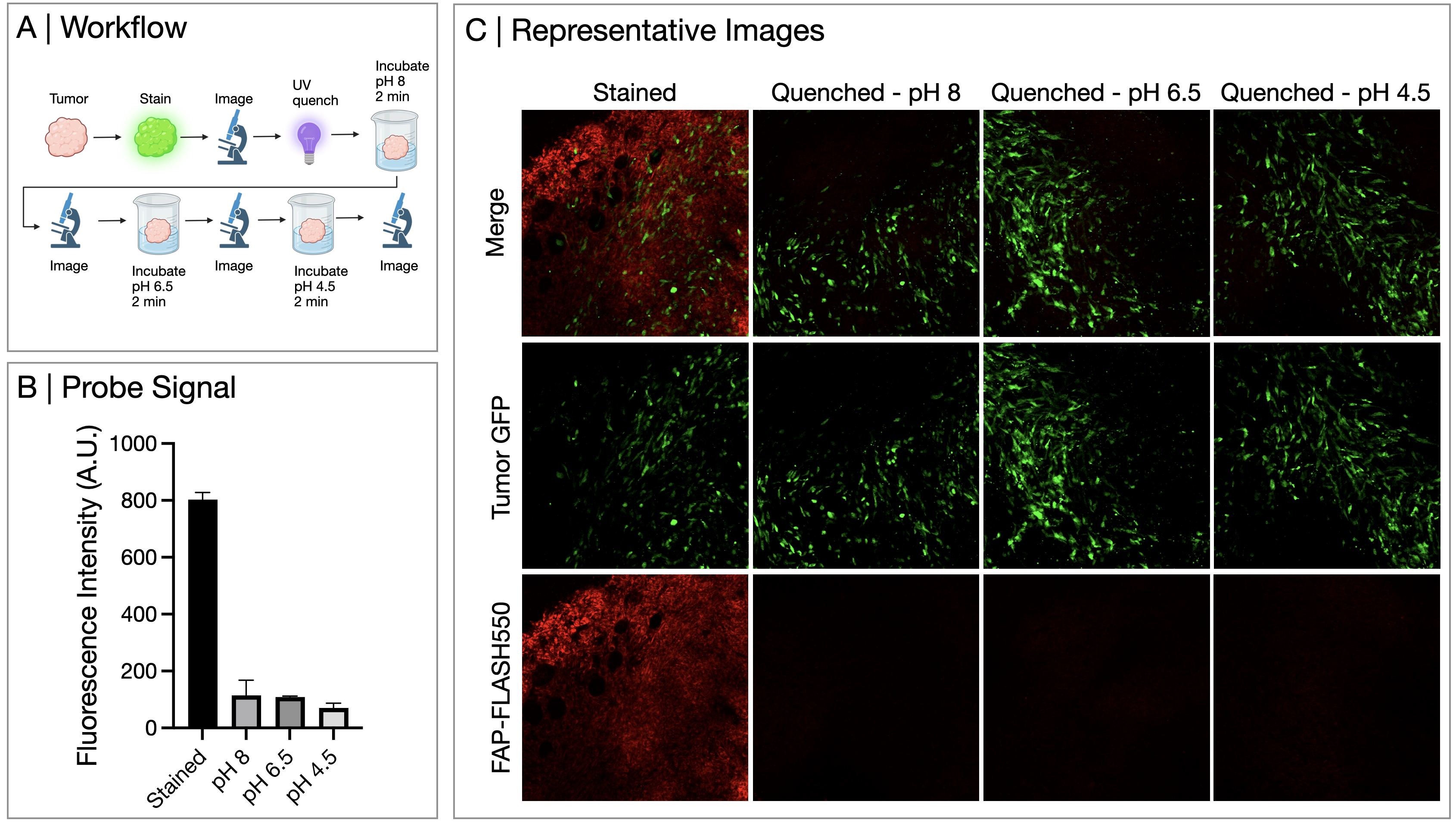


Before UV
